# Brain microstructural alterations in COVID-19: a systematic review of diffusion weighted imaging studies

**DOI:** 10.1007/s11682-026-01084-3

**Published:** 2026-03-14

**Authors:** Ali Jahanshahi, Soheil Mohammadi, Mohammad Amin Salehi, Mahsa Dolatshahi, Sina Mirakhori, Negin Frounchi, Seyed Sina Zakavi, Hamid Harandi, Hosein Ghasempour, Cyrus A. Raji

**Affiliations:** 1 Social Determinants of Health Research Center, Trauma Institute, Guilan University of Medical Sciences, Rasht, Iran; 2 School of Medicine, Guilan University of Medical Sciences, Rasht, Iran; 3 Mallinckrodt Institute of Radiology, Washington University in St. Louis, St. Louis, MO USA; 4 Research Institute, McGill University Health Centre, Montreal, QC Canada; 5 School of Medicine, Tabriz University of Medical Sciences, Tabriz, Iran; 6 Kidney Research Center, Tabriz University of Medical Sciences, Tabriz, Iran; 7 Clinical Research Development Unit of Tabriz Valiasr Hospital, Tabriz University of Medical Sciences, Tabriz, Iran; 8 Research Center for Antibiotic Stewardship and Antimicrobial Resistance, Imam Khomeini Hospital Complex, Tehran University of Medical Sciences, Tehran, Iran; 9 School of Medicine, Tehran University of Medical Sciences, Tehran, Iran; 10 Knight Alzheimer Disease Research Center, Washington University School of Medicine, 4525 Scott Ave., Campus Box 8131, Saint Louis, Missouri 63110 USA

**Keywords:** COVID-19, SARS-CoV-2, DTI, NODDI, Systematic review

## Abstract

**Introduction:**

Following its emergence in Wuhan, COVID-19 has been associated with neurological sequalae, pathophysiological basis of which has been under investigation from the early reports. Herein, we aim to provide a comprehensive overview on white matter microstructural findings in COVID-19 patents.

**Methods:**

We performed a systematic literature search on PubMed, Scopus, Web of Science, and EMBASE databases on February 9th, 2025, using the combination of keywords related to COVID-19, DTI, and NODDI. Study selection and data extraction was performed to provide a qualitative synthesis of the data.

**Results:**

Mean diffusivity (MD) and fractional anisotropy (FA) were the most reported diffusion parameters. Significant alterations in diffusion parameters of longitudinal fasciculi, thalamic radiations, corpus callosum (CC), fronto-occipital fasciculus (FOF), cortico-spinal tract (CST) and uncinate fasciculi (UF) were repeatedly reported among in the studies, of which the results on changes in CR and LF were almost consistent.

**Conclusion:**

The observed changes in white matter microstructural integrity are associated with the psychiatric and cognitive symptoms in post-COVID-19 phase. This observation warrants long-term follow-up of COVID-19 patients for the potential neurological sequalae of this disease.

**Supplementary Information:**

The online version contains supplementary material available at 10.1007/s11682-026-01084-3.

## Introduction

The coronavirus disease 2019 (COVID-19) emerged as a pandemic on January 2020 and has caused 6,974,473 deaths worldwide (WHO Coronavirus, n.d.). Many studies have been conducted to decipher the pathophysiology of this disease, and it is proven that it affects multiple organ systems of the human body (Nalbandian et al. 2021). Regarding the central nervous system (CNS), the complications of COVID-19 vary vastly from vascular and structural effects, such as encephalopathy, strokes, and olfactory or gustatory dysfunctions, to psychiatric and cognitive issues, namely anxiety and mood disorders, sleep disturbances, and dementia (Romoli et al. 2020; Mao et al. 2020; Balcom et al. 2021). Evidence has demonstrated that COVID-19 causes an invasion to neural structures either mediated by immune responses or by direct injury (Desforges et al. 2019). Studies have shown that COVID-19 can infect the CNS through multiple mechanisms. The virus spike proteins have interactions with angiotensin-converting enzyme-2 (ACE-2), which can cause intracellular transmission of the virus via ACE2-specific transmembrane protease serine subtype-2 (TMPRSS2). This interaction in the capillary endothelium can lead to endotheliitis and disruption in the blood–brain barrier. On the other hand, ACE-2 and TMPRSS2 are expressed in the sensory neurons and dorsal root ganglion, which makes them an invasion route for the virus. Moreover, the virus can directly penetrate into the olfactory bulbs, or enter the CNS through lymphatics (Dolatshahi et al. 2021; Lingor et al. 2022). Other mechanisms like delayed immune response, cytokine storms, and oxidative stress are involved in neuronal damage and neurodegeneration (Dolatshahi et al. 2021). Interestingly, Li et al. found that susceptibility and severity of COVID-19 infection is associated with a higher risk of Alzheimer’s disease in a Mendelian randomization study (Li et al. 2022), although the underlying mechanisms are not known. One of the complications of COVID-19 infection is microstructural changes in the CNS, which could potentially underlie the changes in olfaction, cognition, mood, and other neuropsychiatric functions, and possibly higher risk of neurodegenerative diseases (Nalbandian et al. 2021).

Diffusion tensor imaging (DTI) is a widely used MR-based imaging technique that assesses the structural alterations in the CNS according to the diffusion of water molecules in the tracts, which is considered to comply with the normal (Gaussian) distribution. Without any barriers, water molecules would be in the same direction, called isotropic diffusion. However, this can get interrupted by physical obstruction or structural changes, leading to anisotropic diffusion (Basser et al. 1994; Sanjari Moghaddam et al. 2019; Beaulieu 2002). Diffusion parameters such as fractional anisotropy (FA), mean diffusivity (MD), axial diffusivity (AD), and radial diffusivity (RD) are used to depict the structural formation of a neuron and therefore determine the white matter (WM) integrity. FA is a measure of diffusion intensity and is highly sensitive to microstructural alterations. In contrast, MD can reflect changes in diffusion caused by barriers such as cell membrane, and is sensitive to cellular injuries (Clark et al. 2011). AD and RD reflect the diffusion of water molecules in parallel or perpendicular direction, respectively. A decreased AD might be due to axonal damage, while abnormal or diminished myelination increases RD (Sanjari Moghaddam et al. 2019; Alexander et al. 2007; Pierpaoli and Basser 1996; Feldman et al. 2010). DTI data analysis is performed through multiple methods. In Tract-Based Spatial Statistics (TBSS), diffusion information are mapped to a mean FA skeleton in a common space. Then, group-level diffusion metrics are calculated based on these mappings (Smith et al. 2006). In deterministic tractography, after choosing regions of interest (ROI), the pathways connecting these regions are identified and diffusion metrics are estimated (Catani et al. 2002; Wakana et al. 2004). In probabilistic tractography, probable pathways between different points in a network are estimated and the neural networks are reconstructed (Behrens et al. 2007). Network analysis considers brain regions as nodes and WM tracts as connections, where integrity of pathways can be evaluated using network measures such as degree centrality, betweenness centrality, and clustering coefficient (Sporns 2011).

Water diffusion in biologically complex tissues such as brain, does not necessarily follow the normal Gaussian distribution due to multiple obstacles. Diffusion kurtosis imaging (DKI) is a technique that applies a model for DTI analysis, which is more adherent to non-Gaussian distribution. This model has paved the way to evaluate microstructural complexity of the CNS, especially in neurodegenerative disorders (Arab et al. 2018; Nygaard et al. 2025).

Beyond the DTI, neurite orientation dispersion and density imaging (NODDI) is a diffusion MRI model, which gives out promising results on brain microstructure. Based on the tissue compartments in the CNS, namely intracellular, extracellular, and cerebrospinal fluid (CSF), different models of mapping according to the NODDI parameters can be conducted. Neurite density index (NDI) shows the density of axons and dendrites in a voxel and is calculated based on intra-cellular restricted diffusion. On the other hand, apparent fiber density (AFD) is another measure, based on diffusion characteristics and orientation, for estimating fiber density in a voxel. Orientation dispersion index (ODI) is a measure of hindered diffusion in the extracellular compartment. Isotropic volume fraction demonstrates the isotropic diffusivity in the CSF compartment. Studies have shown that in demyelination and neurodegenerative process NDI decreases and ODI increases, displaying a diminished neural connectivity and integrity in fibers (Zhang et al. 2012; Sacco et al. 2020; Preziosa et al. 2023; Bispo et al. 2022).

Performing diffusion-based MRI techniques on COVID-19 patients, different studies have been trying to illuminate the pathophysiology of this pandemic in the CNS. This systematic review delves deeply into this issue and reviews the studies investigating the white matter microstructural changes in COVID-19 patients in order to add knowledge about the neuropsychiatric basis of this disease.

## Methods

We conducted a systematic literature review according to Preferred Reporting Items for Systematic Reviews and Meta-Analyses (the PRISMA 2020 statement) guidelines (Page et al. 2021) and registered the protocol on the International Prospective Register of Systematic Reviews (PROSPERO) website with registration no. of 339,864.

Our purpose was to systematically review the studies assessing microstructural neural changes in COVID-19 patients utilizing the DTI, NODDI, and DKI techniques. A two-step screening process was performed to include eligible studies and any discrepancies were resolved through discussion.

### Search strategy and study selection

We conducted a systematic literature search on PubMed, Scopus, Web of Science, and EMBASE databases on February 9th, 2025, using keywords related to COVID-19, DTI, NODDI, and DKI to find the relevant articles. The search string is illustrated in the supplementary Table 1. After removing duplicate articles, title and abstract screening of the retrieved papers was performed by two reviewers independently (SM and NF). Then a comprehensive full-text screening was conducted independently by two reviewers (SM and NF). Any conflicts were resolved through discussion.

### Inclusion/Exclusion criteria

We included all published case–control or cohort studies assessing microstructural alterations in COVID-19 patients with DTI, NODDI, and DKI techniques until February 9th, 2025, with at least three cases and with no constraint on their language, publication time, and age of participants. Animal studies, conference papers, letters, reviews, and case reports were excluded.

### Data extraction

Two reviewers (NF and SSZ) extracted the following data from the included studies: First author’s name, publication year, study design, study groups and what they were matched for, number of participants, sex distribution, age, and years of education in each group, the diagnostic criteria used for COVID-19, the period of time between the disease and the imaging, method of imaging analysis, DTI measurements such as FA, MD, AD, RD, and NODDI metrics if applicable, mental status of participants in each group, and correlation between cognitive findings and DTI and NODDI parameters. Any discrepancies in this section were resolved with the supervision of the third reviewer (SM).

### Quality assessment

The quality of the included studies was assessed based on the Newcastle–Ottawa scale (NOS) for nonrandomized studies in the domains of sample selection, comparability of the groups, and ascertainment of the exposure, with a maximum score of 4, 3, and 2, respectively. Also, risk of bias for each study was evaluated according to the approach introduced by Viswanathan et al. (2008). In the method introduced in this paper, the risk for each selection, performance, attrition, detection, and reporting bias is assessed by answering a related question. Any discrepancies in quality assessment were also solved through discussion with a third reviewer (MAS).

## Results

### Study selection

**Fig. 1:**
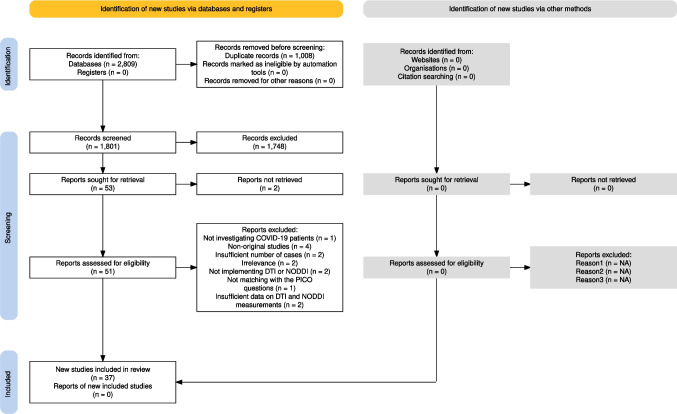
The flowchart of study selection

Our comprehensive search yielded 2809 studies, which were exported to the Endnote software. After removing duplicates, 1801 studies were screened based on their title and abstract. Among them 53 studies were sought for retrieval, of which 51 retrieved for full text screening. The reasons for exclusion were as follows: not investigating COVID-19 patients (n = 1), non-original studies (n = 4), insufficient number of cases (n = 2), irrelevance (n = 2), not implementing DTI, NODDI, or DKI (n = 3), not matching with the PICO questions (n = 1), insufficient data on DTI, NODDI, or DKI measurements (n = 2). Finally, 36 studies met our inclusion criteria and were included for data extraction (Bispo et al. 2022; Benedetti et al. 2021; Huang et al. 2022; Huang et al. 2023; Lu et al. 2020; Qin et al., 2021; Silva et al., 2021; Tian et al., 2022; Yang et al. 2021; Campabadal et al. 2023; Yildirim et al. 2022; Díez-Cirarda et al. 2022; Paolini et al. 2023; Pelizzari et al. 2022; Liang et al., 2023; Qin et al. 2024; Chaganti et al. 2024; 2024; Lipton et al. 2024; Petersen et al. 2023; Arrigoni et al. 2024; Fineschi et al. 2024; Nelson et al. 2024; Sun et al. 2025; Churchill et al. 2024; Ibrahim et al. 2024; Teller et al. 2023; Balsak et al. 2023; Scardua-Silva et al. 2024; Boito et al. 2023; Kausel et al. 2024; Deuter et al. 2024; Trufanov et al. 2025; Planchuelo-Gómez et al. 2023; Mishra et al. 2024; Lith et al. 2024). The flow diagram (Fig. 1) details the study selection process.

### Study characteristics

As shown in Table 1**,** of the total 37 studies, 30 were case-controls (Bispo et al. 2022; Huang et al. 2022; Huang et al. 2023; Qin et al., 2021; Silva et al., 2021; Tian et al., 2022; Yang et al. 2021; Campabadal et al. 2023; Yildirim et al. 2022; Díez-Cirarda et al. 2022; Pelizzari et al. 2022; Liang et al. 2023; Qin et al. 2024; 2024; Lipton et al. 2024; Petersen et al. 2023; Arrigoni et al. 2024; Fineschi et al. 2024; Nelson et al. 2024; Sun et al. 2025; Churchill et al. 2024; Ibrahim et al. 2024; Teller et al. 2023; Balsak et al. 2023; Scardua-Silva et al. 2024; Boito et al. 2023; Trufanov et al. 2025; Planchuelo-Gómez et al. 2023; Mishra et al. 2024; Lith et al. 2024). Except for the study by Benedetti et al. (Benedetti et al. 2021), all of the studies included a control group for comparison. Eight were performed in China (Huang et al. 2022; Huang et al. 2023; Lu et al. 2020; Qin et et al. 2021; Tian et al. 2022; Yang et al. 2021; Qin et al. 2024; Sun et al. 2025). Diffusion imaging was done in all studies, among which eight (Bispo et al. 2022; Huang et al. 2022; Huang et al. 2023; Lipton et al. 2024; Churchill et al. 2024; Ibrahim et al. 2024; Teller et al. 2023; Lith et al. 2024) performed NODDI analysis. All studies except Lipton et al. (Lipton et al. 2024) performed DTI analyses. All of the DTI studies reported at least one of the diffusion measurements of FA, AD, RD, and MD, except for Tian et al. (Tian et al. 2022) who only reported fiber tract volume, and Yildirim et al. (Yildirim et al. 2022) who used quantitative anisotropy for assessing connectivity. Nineteen studies conducted whole-brain TBSS analysis (Benedetti et al. 2021; Huang et al. 2022; Huang et al. 2023; Yang et al. 2021; Díez-Cirarda et al. 2022; Paolini et al. 2023; Pelizzari et al. 2022; Qin et al. 2024; Chaganti et al. 2024; 2024; Fineschi et al. 2024; Nelson et al. 2024; Teller et al. 2023; Scardua-Silva et al. 2024; Boito et al. 2023; Kausel et al. 2024; Deuter et al. 2024; Planchuelo-Gómez et al. 2023; Lith et al. 2024), while Campabadal et al. performed TBSS analysis only for regions of interest (Campabadal et al. 2023). Moreover, seven studies carried out region of interest analysis (Huang et al. 2022; Huang et al. 2023; Liang et al. 2023; Chaganti et al. 2024; Lipton et al. 2024; Balsak et al. 2023; Deuter et al. 2024), of which Huang and colleagues (2022, 2023) and Liang et al. performed post-hoc region of interest analysis based on the TBSS results, as well (Huang et al. 2022; Huang et al. 2023; Liang et al. 2023). Bispo et al. (Bispo et al. 2022) also performed a tract-wise analysis based on fiber density mapping and calculated the parameters such as tissue RD (RDt), and tract average metrics. Six studies (Bispo et al. 2022; Qin et al., 2021; Tian et al., 2022; Yildirim et al. 2022; Arrigoni et al. 2024; Deuter et al. 2024) carried out probabilistic, and other six (Silva et al. 2021; Yang et al. 2021; Sun et al. 2025; Ibrahim et al. 2024; Trufanov et al. 2025; Mishra et al. 2024) performed deterministic tractography.

**Table 1:** Participants’ characteristics of included studies

Study		Country	Study design	Study groups	Diagnostic criteria	N/male	Age (years)	Imaging timeline	Matched for	Education (years)
**Campabadal/2022**	Case	Spain	Case–control	COVID-19 patients with olfactory dysfunction	medical report or biological diagnosis	23/3	51.96 ± 7.92	11.04 ± 3.72 months	-	14.57 ± 2.57
	Control			COVID-19 patients with normal olfaction		25/7	48.04 ± 7.59	8.92 ± 3.72 months		14.72 ± 2.59
**Bispo/2022**	Case	Brazil	Case–control	COVID-19 positive group	RT-PCR	56/20	37.2 ± 9.4	93.3 ± 26.4 days	Age, sex, education	15.3 ± 3.3
	Control			HC		37/15	40.2 ± 11.8			15.0 ± 3.3
**Huang/2023**	Case	China	Case–control	Recovered COVID-19 patients	RT-PCR	17/9	54.82 ± 10.24	2 years	Sex	12
	Control			HC		13/1	51.46 ± 12.61			12
**Díez-Cirarda/2022**	Case	Spain	Case–control	Recovered COVID-19 patients	PCR	86/28	50.71	11.08 ± 4.47 months	Age	14.2
	Control			HC		36/14	49.33			15.43
**Paolini/2022**	Case	Italy	ongoing prospective cohort	With cognitive complaints	RT-PCR	29/18	50.27 ± 13.13	173.12 ± 174.03 days	Age, sex, onset to MRI time, BMI, depressive symptoms	-
	Control			Without cognitive complaints		29/23	54.41 ± 9.93			-
**Pelizzari/2022**	Case	Italy	Case–control	Recovered COVID-19 patients	RT-PCR	22/9	45.7	7.3 months	Age, sex	-
	Control			HC		21/6	37.6			-
**Tian/2022**	Case	China	Case–control	Recovered COVID-19 patients (from 10 months after 3 months follow up)	PCR	MG: 13/6 SG:21/10	MG:58.15 ± 5.67 SG:62.76 ± 5.36	202.31 ± 14.42 days	Age, sex, and education	MG: 11.31 ± 3.43 SG: 11.24 ± 2.76 ‘
	Control			HC		31/18	60.58 ± 6.42			HC: 10.48 ± 3.51
**Qin/2021**	Case	China	Case–control	Recovered COVID-19 patients	PCR	MG:19/7 SG:32/16	MG:59.37 ± 5.87 SG:63.19 ± 5.37	101.21 ± 12.24 days	Age, sex, and education	MG: 11.05 ± 3.12 SG: 10.48 ± 2.67
	Control			HC		31/18	60.58 ± 6.42			10.48 ± 3.51
**Huang/2021**	Case	China	Case–control	Recovered COVID-19 patients	RT-PCR	22/11	54.14 ± 9.76	351.5 days	Age, sex, BMI, education	12
	Control			HC		21/5	49.14 ± 12.44			12
**Benedetti/2021**		Italy	Cohort	Recovered COVID-19 patients without brain lesions	PCR	42/29	54.86 ± 7.89	90.59 ± 54.66 days	-	-
**Yang/2021**	Case	China	Case–control	Recovered COVID-19 patients	PCR and IgG antibodies	28/12	40 ± 7.9	3 months	Age, sex, education, mental health score	15
	Control			HC		27/13	37.7 ± 9.0			15
**Yildrim/2021**	Case	USA	Case–control	COVID-19 related olfactory dysfunction (1.5 months of onset)	PCR	31/10	32.5 ± 10.8	1.5 months for COVI D-19 related olfactory dysfunction, 6 months post-infectious olfactory dysfunction	-	-
	Control			Patients with post-infectious olfactory dysfunction (6 months of onset) including 81.4% with anosmia,and 18.6% with hyposmia		97/38	45.9 ± 13.5			-
**Scardua Silva/2020**	Case	Brazil	Case–control	Recovered COVID-19 patients	PCR/IgM or IgG antibodies	86	-	16–120 days	Age, sex	15
	Control	China	Cohort	HC		133	-			-
**Lu/2020**	Case	China	Cohort	Recovered COVID-19 patients	PCR	60/34	44.10 ± 16.00	97.46 ± 8.01 days	Age, sex	-
	Control	China		HC		39/22	44.10 ± 16.00			-
**Liang/2023**	Case	USA	Case–control	participants with post-COVID-19 conditions	PCR or antigen test	23/8	44.1 ± 12.2	182(42–484) days	Age, sex, education level, and race	-
	Control			HC		24/11	44.3 ± 12.5			-
**Qin/2024**	Case	China	Case–control	Acute Covid with sleep disorder	Not Mentioned	18/29	46.17 ± 15.81	180 days	Age, sex, and education	12.79 ± 3.04
				Acute Covid without sleep disorder		20/27	41.93 ± 16.28			12.26 ± 4.963
	Control			HC		22/30	47.33 ± 15.98			13.57 ± 2.27
**Chaganti/2024**	Case	Austria	Cohort	Participants with post-acute sequelae of SARS-COV-2 infection (PASC) with cognitive impairment	Not Mentioned	14/6	49 ± 2	12 ± 1 weeks	Age, sex	-
	Control			HC		10/5	46 ± 1.7			-
**Serrano del pueblo/2024**	Control	Spain	Case–control	COVID-19 recovered	PCR or antigen test	22/14	50.50 ± 2.59	15 ± 2 month	Age, sex, and education	4.95 ± 0.53
**Lipton/2024**	Case	USA	Case–control	Patients with mild Covid history	clinical- epidemiological criteria	5/2	37 (25–56)	Nm	Nm	-
	Control			HC		15/7	36 (24–57)			-
**Petersen/2023**	Case	Germany	Case–control	Post COVID patients	PCR	223/123	55.54 ± 7.07	289(163,318)	Age, sex, education and cardiovascular risk factors	15.70 ± 2.56
	Control			HC		223/130	55.74 ± 6.60			15.67 ± 2.86
**Arrigoni/2024**	Case	Italy	Case–control	Covid patients with cognitive impairment	RT-PCR	16/6	56[51–61]	293[212–367]	Age, sex	-
				Covid patients with olfactory disorder		35/10	40[31–53]	251[208–286]		-
	Control			HC		14/6	62[45–70]	-		-
**Fineschi/2024**	Case	Sweden	Case–control	Post covid patients	PCR	36/12	44.2 ± 10.2	At least 3 month	Age	-
	Control			Post covid patients		36/12	44.6 ± 10.5			-
**Nelson/2024**	Case	Canada	Case–control	Long covid	PCR or rapid antigen test	56/22	46.16 ± 14.31	6 ± 4 month	Age, sex	14.82 ± 2.35
	Control			Normal covid recovery		35/11	40.83 ± 17.68			15.46 ± 2.11
**Sun/2025**	Case	China	Case–control	Uninfected MDD patients	Virus nucleic acid test	165/47	38.76 ± 11.94	Not mentioned	Age, sex	12.15 ± 3.77
				Infected MDD patients		70/20	42.01 ± 14.72			11.90 ± 4.04
	Control			Uninfected HC		108/36	35.40 ± 10.97			15.94 ± 3.71
				Infected HC		57/15	33.39 ± 11.22			16.21 ± 3.85
**W.Churchill/2024**	Case	Canada	Case–control	Symptomatic COVID-19 positive individuals	PCR	54/18	41 ± 12	Average 120–150 days	-	16.3 ± 2.2
	Control			HC		14/6	41 ± 14			16.3 ± 2.4
**Ibrahim/2024**	Case	Prague	Case–control	Patients with history of COVID19 and neurological symptoms	Serology test (immunoglobulin M,A,G)	41/9	-	Not mentioned	Age	-
	Control			HC and asymptomatic with a history of infection		HC = 28/15asympto = 25/11	-			-
**Balsak/2023**	Case	Turkey	Case–control	Patients with COVID-19	PCR	74/	-	between 90 and 180 days	-	-
	Control			HC		52/25	41.62 ± 12.17			-
**Teller/2023**	Case	Canada	Case–control	COVID + recovered patients	PCR	39/11	42.1 ± 12.7	Initiation and 3 months follow up	Age, sex	-
	Control			Flu like syndrome COVID -		14/5	43 ± 13.7			-
**Scardua‐Silva/2024**	Case	Brazil	Case–control	Unvaccinated individuals after a mild COVID-19 infection	PCR	97/36	41[37.0–42.0]	mean of 97 days after diagnosis	Age, sex	15.7
	Control			HC		77/24	38.2 [34.0–40.0]			16.9
**Boito/2023**	Case	Sweden	Case–control	Previously hospitalized for COVID-19 and experiencing ongoing symptoms of post-COVID condition	PCR	16/16	60 (41–79 years)	210 days	Age	-
	Control			HC		16/11	58 (46–69 years)			-
**Kausel/2024**	Case	Chile	Cohort	patients with Covid infections	PCR	73	19–65 (mean: 40.1)	2–27 month	-	-
	Control			patients with other respiratory infections		27				-
**Deuter/2024**	Case	Germany	Cohort	Acute ill covid patients	PCR	16/13	53.75	Not Mentioned	Age, sex	-
				Recovered covid19 patients		21/10	39.65			-
	Control			HC		13/6	39.62			-
**Trufanov/2025**	Case	Russia	Case–control	post-COVID sundrome	PCR	24/12	49.16 ± 10.65	4–6 month	-	-
	Control			HC		20/6	42.84 ± 8.93			-
**Planchuelo-Gomez/2023**	Case	Spain	Case–control	patients with persistent headache after covid	RT-PCR	40/11	43.7 ± 10.3	10 months (range 3–20 months)	Age, sex	-
				Covid patients with history of episodic		43/9	40.1 ± 6.4			-
				migraine patients with history of chronic migraine		43/6	41.3 ± 7.1			-
	Control			HC		41/11	41.0 ± 9.6			-
**Mishra/2024**	Case	USA	Case–control	Covid recovered patients	RT-PCR	37/25	33.62 ± 10.42	Within 180 days from PCR positive	-	-
	Control			HC		32/20	32.62 ± 8.19			-
**Lith/2024**	Case	Austria	Case–control	Hospitalized Covid-19 patients	PCR/IgG antibodies	49/33	59.53 ± 12.63	40.0 [30.0, 54.0]	Age, sex	-
	Control			HC		25/13	58.48 ± 10.06			-

The participants of sixteen studies were recovered patients (Benedetti et al. 2021; Huang et al. 2022; Huang et al. 2023; Lu et al. 2020; Qin et al., 2021; Silva et al. 2021; Tian et al. 2022; Yang et al. 2021; Díez-Cirarda et al. 2022; Pelizzari et al. 2022; 2024; Lipton et al. 2024; Nelson et al. 2024; Teller et al. 2023; Deuter et al. 2024; Mishra et al. 2024). In three studies conducted by Yildirim et al., Campabadal et al., and Arrigoni et al. participants were included based on their post-infectious olfactory dysfunction (OD) (Campabadal et al. 2023; Yildirim et al. 2022; Arrigoni et al. 2024). Other two included participants based on their sleep (Qin et al. 2024) and major depressive disorder (Sun et al. 2025). Furthermore, two studies divided COVID-19 patients into mild and severe subgroups and reported their results separately (Qin et al., 2021; Tian et al. 2022). All of the participants in the studies were in the post-acute COVID-19, except in six studies conducted in acute phase (Qin et al. 2024; Arrigoni et al. 2024; Churchill et al. 2024; Deuter et al. 2024; Planchuelo-Gómez et al. 2023; Lith et al. 2024). The mean age of the participants ranged from 32.5 ± 10.8 (Yildirim et al. 2022) to 63.19 ± 5.37 (Qin et al. 2021). All of the studies, except fourteen (Benedetti et al. 2021; Huang et al. 2023; Campabadal et al. 2023; Yildirim et al. 2022; Díez-Cirarda et al. 2022; Lipton et al. 2024; Fineschi et al. 2024; Churchill et al. 2024; Ibrahim et al. 2024; Balsak et al. 2023; Boito et al. 2023; Kausel et al. 2024; Trufanov et al. 2025; Mishra et al. 2024), included an age and sex-matched control group, although in four studies (Díez-Cirarda et al. 2022; Fineschi et al. 2024; Ibrahim et al. 2024; Boito et al. 2023), the groups were only matched for their age.

The primary diagnostic approach for COVID-19 was a PCR test in almost all studies. However, five studies (Silva et al. 2021; Yang et al. 2021; Campabadal et al. 2023; Ibrahim et al. 2024; Lith et al. 2024) made their diagnosis based on an antibody assay and medical or biological criteria. All studies reported an imaging timeline representing the time between the discharge of the patient and the MRI image acquisition, which ranged from 16 days (Silva et al. 2021) to 27 months (Kausel et al. 2024).

### Quality assessment and publication bias

All of the cohort studies, except for one (Kausel et al. 2024), obtained a score of 9 according to the NOS scale (Benedetti et al. 2021; Lu et al. 2020; Paolini et al. 2023; Chaganti et al. 2024; Deuter et al. 2024), and none matched the participants for education. All of the case–control studies obtained complete scores in selection and exposure domains. In the comparability domain, six case–control studies did not match groups for age, sex, or education (Campabadal et al. 2023; Yildirim et al. 2022; Lipton et al. 2024; Churchill et al. 2024; Balsak et al. 2023; Trufanov et al. 2025; Mishra et al. 2024), and eight studies gained the full score of 3 in this domain matching the groups for age, sex, and education (Huang et al. 2022; Qin et al. 2021; Tian et al. 2022; Yang et al. 2021; Liang et al., 2023; Qin et al. 2024; Serrano et al. 2024; Petersen et al. 2023).

Regarding risk of bias, all of the studies, except for one (Balsak et al. 2023), were low risk in the attrition and reporting bias domains. Moreover, all except two (Benedetti et al. 2021; Yildirim et al. 2022) were low risk in the selection domain. More detailed information on quality assessment and risk of bias is illustrated in Supplementary Table S2 and Supplementary Table S3.

### Between-group differences

#### Cortical regions

Three studies mentioned alterations in the orbitofrontal and entorhinal regions (Campabadal et al. 2023; Yildirim et al. 2022; Lipton et al. 2024). Yildirim et al. found that quantitative anisotropy in patients with post-infectious olfactory dysfunction was higher in orbitofrontal and entorhinal regions and connections in these areas (Yildirim et al. 2022). Campabadal et al. (Campabadal et al. 2023) noted an increased MD in the orbitofrontal WM in patients with olfactory dysfunction. Furthermore, Lipton and colleagues (Lipton et al. 2024) found a decreased volume fraction of isotropic diffusion compartment (V_iso_) in the right middle orbitofrontal WM and a decreased ODI in the left orbitofrontal regions, and the right entorhinal gray matter.

Benedetti et al. (Benedetti et al. 2021) noted that COVID-19 patients had lower FA and AD and higher MD and RD in the anterior cingulate cortex on both sides. Similarly, Sun et al. and Mishra et al. (Sun et al. 2025; Mishra et al. 2024) found a reduction in FA in the left and right cingulum cingulate, respectively. Besides, Mishra et al. reported a decreased AD and RD in the same region (Mishra et al. 2024). Additionally, Bispo et al. (Bispo et al. 2022) reported that AFD was lower in the cingulate gyrus.

Arrigoni and colleagues (Arrigoni et al. 2024) mentioned the highest alteration in gray matter MD in the right precentral, as well as both frontal and parietal regions. Moreover, based on the network analysis performed by Yang and colleagues (Yang et al. 2021), in COVID-19 patients, local and global efficiencies were lower, and the shortest path length was longer in the superior occipital region.

#### White matter tracts

There were 25 studies reporting specific results on WM clusters (Bispo et al. 2022; Benedetti et al. 2021; Huang et al. 2023; Lu et al. 2020; 2021; Tian et al. 2022; Yang et al. 2021; Campabadal et al. 2023; Díez-Cirarda et al. 2022; Paolini et al. 2023; Pelizzari et al. 2022; Liang et al. 2023; Qin et al. 2024; Chaganti et al. 2024; 2024; Petersen et al. 2023; Arrigoni et al. 2024; Sun et al. 2025; Churchill et al. 2024; Ibrahim et al. 2024; Balsak et al. 2023; Deuter et al. 2024; Trufanov et al. 2025; Planchuelo-Gómez et al. 2023; Mishra et al. 2024), among which Tian and colleagues and Deuter et al. reported comprehensive results on volume (Tian et al. 2022; Deuter et al. 2024). Nine studies found significant differences between COVID-19 and control groups in thalamic radiations (Bispo et al. 2022; Huang et al. 2023; Tian et al. 2022; Yang et al. 2021; Campabadal et al. 2023; Pelizzari et al. 2022; Sun et al. 2025; Trufanov et al. 2025; Lith et al. 2024), two of which (Yang et al. 2021; Sun et al. 2025) reported a decreased FA in the left thalamic radiations. Incongruently, Trufanov et al. (Trufanov et al. 2025) noted that FA was increased in the left thalamic radiations. Campabadal et al. (Campabadal et al. 2023) and Pelizzari et al. (Pelizzari et al. 2022) reported higher MD in the anterior and higher RD in the superior thalamic radiation, respectively. Eight studies mentioned the corticospinal tract (CST) in their results (Bispo et al. 2022; Huang et al. 2023; Silva et al. 2021; Tian et al. 2022; Pelizzari et al. 2022; Qin et al. 2024; Trufanov et al. 2025; Planchuelo-Gómez et al. 2023). Silva et al. (2021) detected an elevated FA and AD, and Pelizzari and colleagues (Pelizzari et al. 2022) found an increased RD in the CST. Similarly, Qin et al. (Qin et al. 2024) reported increased RD in CST in COVID-19 individuals with sleep disorders. They also noted lower FA and AD. Consistent with these, Trufanov and colleagues (Trufanov et al. 2025) mentioned lower FA in the right CST. Detailed information is mentioned in Table 2.

**Table 2:** Overview of the changes in diffusion parameters

Study	Study group	Diffusion model	Field Strength/ b value (s/mm2)	Method of Analysis: Studied Tracts/Regions	DTI results			
					**FA**	**MD**	**AD**	**RD**
**Campabadal/2022**	Patients with persistent olfactory dysfunctions after COVID-19	DTI	3 T/ 3000	**TBSS:** Amygdalae, hippocampi, insular cortices, olfactory cortex, parahippocampal gyri, gyrus rectus, the genu of CC, external capsule, the uncinate fasciculi, and the anterior thalamic radiation	No significant differences	Higher MD values in the orbitofrontal WM tracts, genu of the CC, the forceps minor, and the anterior thalamic radiation	No significant differences	Higher RD values in the genu of the CC, anterior CR, and uncinate fasciculus
**Bispo/2022**	COVID-19 patients at least 4 weeks after diagnosis	DTI, NODDI	3 T/800	**TractoFlow was used for extracting DTI and CSD measures:** **1) Voxel-based analysis**: to investigate the metrics FW, FAt, MDt, RDt, ADt, and AFDtotal, extracted from the fiber orientation distribution function (Fixel-based analysis by separating the extracellular FW component from the “tissue” component), **2) Whole brain probabilistic tractography,** **3) Tractometry of each bundle using the FD**	No significant differences	No significant differences	Reduced tissue AD in the right arcuate fasciculus	Increased tissue RD in the left SLF
**Huang/2023**	Recovered COVID-19 patients	DTI, NODDI	3 T/-	**TBSS:** Whole-brain voxel-wise analysis **Post hoc region of interest analysis:** for regions with significant results on TBSS	-	-	-	Increased PT2 (two years after discharge) than in PT1 (a year after discharge)
**Diez-cirard 2022**	Post-COVID-19 syndrome patients	DTI	3 T/-	**TBSS:** Whole-brain voxel-wise analysis	Reduced in whole brain	-Reduced in whole brain - Lower in CC, middle longitudinal fasciculus, forceps minor and uncinate tract and fronto-occipital fasciculus -Increased in hospitalized patient compare to non-hospitalized	-Reduced in whole brain - Lower in CC, middle longitudinal fasciculus, forceps minor, uncinate fasciculus and fronto-occipital fasciculus	Reduced in whole brain
**Paolini 2022**	Recovered COVID-19 patients with subjective cognitive impairments	DTI	3 T/ 1000	**TBSS:** Whole-brain voxel-wise analysis	No significant difference, but cognitive complainers show marginal trend of lower values at p_FWE_ = 0.09	Higher in wide-range areas of WM regions on both sides, such as IFOF, uncinate fasciculus, CR and multiple sections of CC. Increased MD correlated with increased RD in left hemisphere and greater AD in some inter-hemispheric associative tracts	Higher in some inter-hemispheric associative tracts, associated with increased MD	Higher in many WM tracts in the left hemisphere (CR, inferior longitudinal fasciculus, IFOF, SLF and uncinate fasciculus), correlated with increased MD
**Pelizzari/2022**	Recovered COVID-19 patients	DTI	3 T/ 1000	**TBSS:** Whole-brain voxel-wise analysis	Decreased in right arcuate fasciculus, right middle longitudinal fasciculus, right SLF II, right SLF III	No significant difference	No significant difference	Increased in right arcuate fasciculus, acoustic radiation, dorsal cingulum, CST, frontal aslant tract, IFOF, middle longitudinal fasciculus, optic radiation, SLF I, SLF II,superior longitudinal fas- ciculus III, and superior thalamic radiation
**Rau 2022**	Subacute COVID-19 patients	DMI (Calculation of parameters by a supervised machine learning approach, i.e. a Bayesian estimator)	3 T/ 1000 & 2000	**Voxel-wise analysis: **Whole brain Streamline-wise analysis: “sparse” reconstruction setting with 5000 streamlines, the V-CSF values of every voxel attached to a particular streamline were averaged to obtain a streamline-specific value	-	-	-	-
**Tian 2022**	Recovered mild (MG) and severe (SG) COVID-19 patients after 10 months (MG2, SG2) and 3 months (MG1, SG1) follow up	DTI	3 T/1000	**Probabilistic tractography and XTRACT pipeline for automatic extraction of fiber tracts:** volume as a measure of interest	-	-	-	-
**Qin 2021**	Recovered COVID-19 patients	DTI	3 T/1000	**Probabilistic tractography using XTRACT software package (using BEDPOSTX and PROBTRACKX tractography protocols)** Tracts similar to Tian2022	MG and SG compared with NC in association, commissural, projection, and limbic fiber bundles show a reduction in mean FA -	-	-	-
**Huang/2021**	Recovered COVID-19 patients	DTI, NODDI	3 T/ 1000 & 2000	**TBSS**: Whole-brain voxel-wise analysis **Post-hoc region of interest**: For significant regions is TBSS	Lower in the body of the CC in the ICU group than in the non-ICU group	-	-	-
**Benedetti/2021**	COVID-19 survivors without brain lesion	DTI	3 T/1000	**TBSS:** Whole-brain voxel-wise analysis	-	-	-	-
**Yang/2021**	Recovered COVID-19 patients	DTI	3 T/1000	**TBSS**: Whole white matter network **Fiber Assignment by Continuous Tracking (or FACT): Deterministic DTI tractography** and network analysis	-Decreased in the CC, CR, internal capsule, external capsule, SLF, left posterior thalamic radiation, left cingulum, and left superior fronto-occipital fasciculus	-Increased values in similar regions to FA reduction	No significant differences	-Increased values in similar regions to FA reduction
**Yildirim/2021**	Patients with persistent COVID-19 related olfactory dysfunction (OD)	DTI	3 T/1000	**Tractography (probabilistic):** Thirty-six tracts between right orbitofrontal and entorhinal field and 13 tracts between left orbitofrontal and entorhinal field	-	-	-	-
**Silva/2020**	Post-COVID-19 patients	DTI	3 T/1000	**Tractography (deterministic):** Commissural tracts (CC divided into three parts: Genu, body and splenium), association tracts (inferior longitudinal fasciculus, IFOF and uncinate fasciculus), limbic tracts, dorsal cingulum and parahippocampal cingulum) and one projection tract (CST)	-Increased: Genu of CC, CSTs, Dorsal Cinguli, Parahippocampal Cinguli, Uncinate Fasciculi, Inferior longitudinal Fasciculi -No statistically significant difference: Body of CC, Splenium of CC, Inferior Occipital Fasciculi	-No statistically significant difference: Genu of CC, Body of CC, CSTs, Dorsal Cinguli, Inferior longitudinal fasciculi,Inferior occipital fasciculi -Increased: Splenium of CC, Parahippocampal Cinguli -Decreased: Uncinate fasciculi	-No statistically significant difference: Genu of CC, Body of CC, Uncinate fasciculi, Inferior longitudinal fasciculi, Inferior occipital fasciculi -Increased: Splenium of CC, CSTs, Dorsal Cinguli, Parahippocampal Cinguli	-No statistically significant difference: Genu of CC -Body of CC -Splenium of CC, CSTs, Dorsal Cinguli, Inferior longitudinal fasciculi, Inferior occipital fasciculi -Increased: Parahippocampal Cinguli-Decreased: Uncinate fasciculi
**Lu/2020**	Recovered COVID-19	DTI	3 T/ 1000	Extracting Brain regional volumes, and diffusion indices including FA, MD, RD and AD of WM and GM from normalized DTI quantitative maps by rigid registration between the DTI quantitative maps and 3D-T1WIs -Global analyses as well as regional analyses3D-T1WIs based parcellation using 65 regions of anatomical labelling atlas-3 (AAL-3) and 36 regions of JHU DTI-based White Matter (WM) atlas	-Considerably higher global FA value -Higher mean regional FA in the white matter -No notable differences in regional FA	-Lower global MD -Considerably lower MD values in the gray matter of the left insula, bilateral cingulate gyri, right precuneus, and right thalamus -Lower mean regional MD values in the white matter -Significantly lower mean MD values of right superior frontal-occipital fasciculus	-Lower global AD -Lower mean regional AD in the white matter -Considerably lower AD values in right CR, right external capsule and right superior frontal-occipital fasciculus	-Lower global RD -Generally lower mean regional RD in the white matter -No significant differences in regional RD **Global Analysis:**
**Liang/2023**	participants with post-COVID-19 conditions (PCC)	DTI	3 T/1000	**ROI:** FA, MD, RD, AD values were obtained for 9 major white matter fiber tracts (corpus callosum, corona radiata, internal capsule, external capsule, cingulum, sagit- tal stratum, fronto-occipital fasciculus, longitudinal fasci- culus, uncinate fasciculus) and MD for 6 subcortical grey matter regions (amygdala, hippocampus, caudate nucleus, putamen, globus pallidus, thalamus) **Post-hoc analyses:** sub-regions when there was a significant/trend level group difference on effects of interest in the main region	-PCC participants had higher sagittal stratum FA bilaterally -PCC participants had higher superior longitudinal fasciculus FA	-PCC participants showed lower MD compared to controls. -PCC women had higher MD than control women. -No significant MD difference between PCC and control men	-	-PCC participants had lower right sagittal stratum RD -PCC participants had lower right superior longitudinal fasciculus RD
**Qin/2024**	Covid19 patient with or without sleep disorders	DTI	Not mentioned	**TBSS:** Whole-brain	-Covid-SD in comparison to HC:significant lower FA in BCC, SCC, right PLIC, left SCR, bilateral PCR, PTR, bilateral ATR, bilateral CST, fmajor, right ILF, and right IFOF -Covid-NSD in comparison to HC show significant lower FA in BCC, SCC, bilateral SCP, right CP, right PLIC, bilateral PCR, bilateral PTR, bilateral ATR, right CST, fmajor, right ILF, and right IFOF -Higher FA in covid-SD compare to covid-NSD in the left CST -Covid-SD group had lower FA in right IFOF		-Significant lower AD of Covid-SD group in BCC, SCC, PLIC, SCR, PCR,PTR, ATR, CST, fmajor, ILF,IFOF -Significant lower AD of Covid-NSD in comparison with HC in in the BCC, SCC, bilateral SCP, right CP, right PLIC, bilateral PCR, bilateral PTR, bilateral ATR, right CST, fmajor, right ILF, and right IFOF -Lower AD in covid-SD in right IFOF	-Significant higher RD of Covid-SD group in BCC, SCC, PLIC, SCR, PCR,PTR, ATR, CST, fmajor, ILF,IFOF -Significant lower AD of Covid-NSD in comparison with HC in in the BCC, SCC, bilateral SCP, right CP, right PLIC, bilateral PCR, bilateral PTR, bilateral ATR, right CST, fmajor, right ILF, and right IFOF -Higher RD in covid-SD in right IFOF
**Chaganti/2024**	post-acute sequelae of SARS-COV-2 infection (PASC) with cognitive impairment (CI)	DTI	3 T/0, 1000	**ROI:** basal ganglia (caudate and lentiform nucleus), frontal cortex, frontal white matter, thalami, splenium of corpus callosum, occipital cortex and white matter, internal capsule, brainstem and cerebellar lobes **TBSS:** whole-brain	-FA increases significantly in PASC CI participants compared to controls in widespread regions (Frontal white matter, corpus callosum, cerebral peduncles, and sagittal striatal white matter)	-Significant MD decrease in PASC CI participants compared to controls in various regions, (Frontal white matter, corpus callosum, cerebral peduncles, and sagittal striatal white matter)		
**Serrano del pueblo/2024**	acquired COVID-19 with persistent neurological symptoms	DTI	1.5 T/-	**vertex-wise analysis:** Whole brain **TBSS:** Whole-brain	-Significant between group FA difference -Significant lower FA of in both hemispheres in the white matter underlying the dorsolateral, orbitofrontal and medial frontal cortices in patients compare to controls -Lower FA in the cingulum bundle, rostrum and genu of the corpus callosum, uncinate fasciculus, superior and inferior longitudinal fasciculus including the white matter of the anterior part of the temporal lobe (temporal stem), parts of the arcuate fasciculus, splenium of the corpus callosum, and medial and lateral occipitotemporal white matter			-Significant between group RD difference -In long Covid patients, no white matter areas showed higher FA or lower RD compared to controls. Instead, areas with higher RD values overlapped with those having lower FA, and these changes were more widespread across the white matter skeleton
**Lipton/2024**	Patients with mild Covid history	NODDI	3 T/0, 300, 800, 2000	**ROI**: regional gray and white matter				
**Petersen/2023**	Post Covid patients	DTI	3 T/ 1000	**vertex- and voxel-wise analyses:** gray and white matter	FA increased in 0.8% of the white matter skeleton and decreased in 1.2% in cases compared to controls FA and tissue FA (FA_T_)also showed significant differences in association and commissural tracts	Significant increase (41.3%) and decreased in 0.1% of the skeleton in cases	-	**-**
**Arrigoni/2024**	Covid patients with olfactory (Covid-OD) or cognitive impairment (Covid-CM)	DTI	3 T/0 and 1000	**tractography** **Tractometry** **Fixed-based analysis (probabilistic):** Whole brain	-Significant FA change in WM of covid-CM at global level.(after adjusting for age, sex and BrainsegVol it was not significant) -Decreased FA of WM in tracts within the medulla in Covid-CM patients, and the middle cerebellar peduncle in the Covid-OD group -Significant FA decrease in the right uncinate fasciculus (UF) in Covid-OD patients	-Significant MD increase in overall GM in both patients group in comparison to HC. (Highest alteration in the left and right frontal area, the right precentral, and both parietal regions. also in covid patients with olfactory disease left precentral and right cuneus GM regions were also involved) -Significant WM MD increase of Covid-CM compare to controls. A significant increase in MD was observed at the WM regional level in the medulla, posterior corona radiata, forceps minor of the corpus callosum bundle, and, similar to the Covid-OD group, in the right inferior and middle cerebellar peduncle -Tractometry and MD regional analysis on major WM bundles showed a significant increase in MD within the forceps minor section of the corpus callosum in COVID-CM patients		
**Fineschi/2024**	Post covid patients	DTI	3 T/ -	**Voxel-based morphometry analysis:** Gray matter **TBSS:** White matter	No significant negative correlation between FA off the right superior-/middle- temporal gyrus and the Symptom Severity Scale			
**Nelson/2024**	Long covid and normal recovered patients	DTI	3 T/700	**TBSS**: whole-brain voxel-wise analysis	no group differences	Significant lower MD in long covid (also long covid group with symptoms length greater than 30 days and long-COVID group has ongoing neurocognitive symptoms) In comparison to normal recovered	no group differences	no group differences
**Sun/2025**	MDD patients infected with covid-19	DTI	3 T/0, 1000	**Tractography**: 20 WM bundle masks (Atlas-based, Deterministic)	-Significant interaction between MDD and covid-19 on FA in the right Cingulum-hippocampus tract -Infected HC group with covid in comparison with uninfected HC, had decreased FA in 13 white matter tracts -Infected MDD group compared to uninfected MDD group, showed decreased FA in left Anterior thalamic radiation, left Cingulum-cingulate gyrus, Forceps minor, and bilateral Superior longitudinal fasciculus -Significant difference of FA value between MDD and HC group in 14 tracts, but no significant difference between uninfected MDD and uninfected HC group			
**W.Churchill/2024**	Post-acute COVID syndrome (PACS)	DTI, NODDI	3 T/700,1400,2100	**Voxel-wise** analyses of the dMRI parameter maps: white matter	No significant differences	Significant decrease of MD in right anterior corona radiata, splenium of the corpus callosum and right posterior corona radiata	Significant decrease on AD in splenium of corpus callosum, including right superior, and medial aspects and left sagittal stratum	No significant effect on RD
**Ibrahim/2024**	Patients with Covid19 history experiencing neurological symptoms	DTI, NODDI	3 T/0 to 4000	**Tractography (deterministic):** Whole brain	Insignificant decrease	significant difference of MD in controls and symptomatic patients in the forceps minor (P = 0.001) and body of the CC (P = 0.003)	Insignificant increase	Insignificant increase
**Teller/2023**	COVID19 + patients	DTI, NODDI	3 T/0, 700, 1400, 2100	**TBSS**		-In the simulation correlated diffusion imaging and MD has inverse correlation		
**Balsak/2023**	patients with COVID-19	DTI	1.5 T/0 and 1000	ROI: bulbus, pons, thalamus, caudate nucleus, globus pallidum, putamen, and hippocampus	-Significant higher FA of bulbs, thalamus putamen in cases compare to healthy controls -Significant higher FA of globus palladium in healthy controls compare to inpatients and outpatients -High FA of putamen in inpatient group than outpatient group -Negative correlation between FA of mentioned areas and plasma LDH			
**Scardua‐Silva/2024**	Unvaccinated individuals after a mild Covid infection	DTI	3 T/ 1000	**TBSS**: white matter	No significant difference	No significant difference	No significant difference	Higher AD in patients
**Boito /2023**	Patient with previously hospitalized for Ccovid and experiencing ongoing symptoms of post-Ccovid condition	DTI	3 T/ 100,700,1400,2000	**TBSS**: brain white matter	FA and microscopic FA was lower in patients white matter	MD and variance in MD was higher in patients	In whole brain of patients RD was higher than AD	AD was higher than RD in patients only in white matter of left frontal lobe
**Kausel/2024**	patients with respiratory infections	DTI	-/ 0 and 1000	**TBSS**: Whole brain	Anosmia was associated with decreased FA in white matter	applying an uncorrected threshold MD increased in frontal and parietal fascicles	applying an uncorrected threshold AD increased in frontal and parietal fascicles	
**Deuter/2024**	patients in acute COVID phase and recovered patients	DTI	1.5 and 3 T/ 0, 1000	**1)Voxel-based morphometry:** whole brain grey matter analysis **ROI:** The gray matter volume **2)Probabilistic tractography:** white matter and subcortical fiber tracts **TBSS:** whole brain	Significant lower FA in acute cases in comparison to controls (in AF bilateral, FAs bilateral, IFO bilateral, MdLF bilateral, OR bilateral, CBD left, CBP left, SLF3 left, VOF left, CBT right, AC, FMI) No significant difference between recovered cases and controls -In TBSS widely reduced FA value in acute cases comparing to recovered and control group, in a variety of fiber tracts	Significant MD difference comparing acute cases and controls (in ATR bilateral, CBT bilateral, FX bilateral, IFO bilateral, MdLF bilateral, AF left, UF left, OR right, AC) -Significant higher MD in acute cases compared to recovered cases (in AF bilateral, ATR bilateral, CBD bilateral, CBP bilateral, CBT bilateral, FAs bilateral, FX bilateral, ILF bilateral, IFO bilateral, MdLF bilateral, OR bilateral, UF bilateral, VOF bilateral, SLF2 left, SLF3 left, AR right, AC, FMI, MCP) -No significant difference of recovered cases and controls (except in SLF2 left) -In TBSS widely increased MD value in acute cases comparing to recovered and control group, in a variety of fiber tracts		
**Trufanov et al./2024**	Post COVID syndrome	DTI	3 T/1000	**Tractography (deterministic):** whole brain	FA increased in left fornix, corpus callosum tapetum, right cerebellum, left inferior fronto-occipital fasciculus, left inferior longitudinal fasciculus, left thalamic radiation superior, and left uncinate fasciculus And decrease in right cerebellum, left cerebellum, corpus callosum forceps major, right corticospinal tract, middle cerebellar peduncle, and right medial lemniscus			
**Planchuelo‐Gómez/ 2023**	patients with persistent headache after Covid, and patients with history of episodic and chronic migraine	DTI	3 T/1000	**TBSS**: Whole-brain	-Lower FA in Covid patient in comparison to HC group in white matter regions like the internal/external capsules, corona radiata, and corpus callosum -Covid group in comparison to migraine groups (EM or CM) had lower FA in bilateral corona radiata and left internal capsule -Comparing migraine and HC group CM had lower FA in regions like the corpus callosum and fornix	Covid group in comparison to migraine groups (EM or CM) had reduced MD in cerebellar/cerebral peduncles -Comparing migraine and HC group revealed lower MD in CM vs EM across cerebellar/cerebral peduncles and corticospinal tracts	- Covid group in comparison to migraine groups (EM or CM) had reduced AD in cerebellar/cerebral peduncles -Comparing migraine and HC group revealed lower AD in CM vs EM across cerebellar/cerebral peduncles and corticospinal tracts	-Higher RD in Covid patient in comparison to HC group in left-hemisphere areas such as the superior longitudinal fasciculus -COV group in comparison to migraine groups (EM or CM) had mixed RD differences (lower in some regions, higher in others like the corpus callosum)
**Mishra/ 2024**	Covid recovered patients	DTI	3 T/0 and 1000	**Tractography (deterministic)**: whole brain (20 white matter tracts analysed)	Decrease FA in left uncinate fasciculus and right cingulum cingulate, increase FA in right cingulum hippocampus	No significant differences in MD	Decrease AD in left arcuate fasciculus and right cingulum cingulate	Increase RD in left uncinate fasciculus and decrease in right cingulum hippocampus
**Lith/ 2024**	hospitalized Covid patients	DTI, NODDI	3 T/ 0 and 1000	**TBSS:white matter tracts**	no significant differences between case and control	no significant differences between case and control		

Study	NODDI results	Other Imaging Findings						
	**V**_**ic**_	**V**_**ec**_	**V**_**iso**_	**V**_**CSF**_	**ODI**	**FD**	**AFD**	
**Campabadal/2022**	-	-	-	-	-	-	-	COVID-19 patients with olfactory dysfunction had less gray matter volume than normal olfactory patients in a group of the following parts: Insular cortex, left amygdala, inferior orbital and frontal superior gyri, parahippocampal gyrus, gyrus rectus, caudate, putamen, and olfactory cortex
**Bispo/2022**	-	-	-	-	-	-In the tract-average analysis, the COVID-19 + group had reduced fiber density in the SLF and left arcuate fasciculus, compared with the COVID-19- group -In tractometry, decreased FD was found in bundle sections within the cingulum, arcuate fasciculus, fornix, inferior & SLF, uncinate fasciculus, IFOF, CST, CR, and CC (rostral body and posterior genu) in the COVID-19 + group in comparison with the controls	- AFD total values were lower in the COVID-19 + group than COVID-19- group. The affected tracts included the CST, left anterior thalamic radiation, IFOF, cingulate gyrus, inferior & SLF, and temporal part of SLF	-The vertex-wise cortical thickness did not differ between COVID-19 + and COVID-19- groups. The thalamus, putamen, pallidum, caudate, hippocampus, accumbens, and amygdala volumes did not differ -No differences were observed between the groups for tissue FA, RD, AD, MD, and FW using TBSS -In the tract-average analysis, no differences were observed between the groups for tissue FA, RD, AD, MD, and FW
**Huang/2023**	-Lower in PT2 compared with PT1	-	-Greater in PT2 compared to HC -Lower in PT2 compared with PT1	-	-Lower in PT2 compared with PT1	-	-	**Diffusion metrics:** Abnormal diffusion metrics in: Posterior thalamic radiation (PTR), sagittal stratum (SS), CC, cerebral peduncle, internal capsule L, PTR, external capsule L, internal capsule L, and CST L, bilateral CR, SLF
**Diez-cirard 2022**	-	-	-	-	-	-	-	**Functional connectivity:** Reduced in particular between left and right parahippocampal gyri, as well as vermis to the left frontal superior orbital cortex and right frontal superior orbital cortex -Reduced in hospitalized patients in the left and right para-hippocampal areas **Gray matter volume:** Reduced in the parahippocampal gyrus, frontal gyrus, anterior cerebellar, occipital lobe and bilateral superior temporal lobe -Reduced in hospitalized patients compare to non-hospitalized **WM Hyperintensities (WMH):** No difference in number of lesions and total lesion volume. However, after adjusting age, significantly higher WMH number and total lesion volume in control group was observed
**Paolini 2022**	-	-	-	-	-	-	-	**Multivoxel Pattern Connectivity analysis:** Different rs-FC in 2 clusters; first in the right frontal pole and second in the middle temporal gyrus **Post-hoc seed-to-voxel:** increased FC in cognitive complainers in 8 clusters (all were parts of the Salience, Dorsal Attention or Sensori-Motor networks): Inferior Lateral Occipital cortex, bilateral Insular Cortex, bilateral Precentral Gyrus, Anterior Cingulate gyrus and bilateral Supramarginal and Opercular cortex. Reduced FC in 5 clusters (all were parts of the Default Mode Network): posterior Cingulate gyrus, right Cerebellum, bilateral superior Occipital cortex, left middle temporal gyrus (MTG). No Network in the left MTG cluster. Last cluster was in cerebellar networks. Second seed: Lower FC in the right Frontal Pole and left posterior MTG. No increased FC
**Pelizzari/2022**	-	-	-	-	-	-	-	**Gray matter Volume:** No significant difference **WM focal lesions:** No significant difference **Brain perfusion:** No significant difference
**Rau 2022**	Decreased	Decreased	Increased	Increased (more prominent in frontal and parietal WM based on streamline analysis)	-	-	-	-
**Tian 2022**	-	-	-	-	-	-	-	***Cortical Thickness:*** -Thicker cortex in theMG2 than the MG1 in the left limbic areas, right parahippocampus, bilateral frontal, and left temporal-parietal cortex -Thicker cortex in the SG2 in comparison with SG1 in the left limbic area and left temporal-frontal cortex + atrophy in right sensorimotor areas and right temporal-parietal cortex -No significant differences were observed in NC-MG2, NC-SG2, and MG2-SG2 comparisons ***CBF comparison:*** -No changes in cortical CBF in MG, SG2 showed extensive lower CBF values than controls, especially in bilateral frontal cortices and temporal cortices, with a reduction in the hypoperfusion areas in the SG2 compared with SG1 - After FDR correction, no surviving results were observed in subcortical nuclei volume and CBF analysis ***White matter differences in comparisons*** **-**MG2 in comparison with MG1 has considerably higher volume in the left anterior thalamic radiation and lower volumes in the right CST and left vertical occipital fasciculus. Also in comparison with NC has significant lower volumes in the left acoustic radiation, right CST, right frontal aslant tract, right inferior longitudinal fasciculus, right middle longitudinal fasciculus, and right vertical occipital fasciculus -SG2 in comparison with SG1 shows significant greater volumes in the right acoustic radiation, right fornix, and right SLF I, as well as lower volumes in the left CST, right superior thalamic radiation, and bilateral vertical occipital fasciculus -SG2 displayed significantly lower volumes than normal control in the right CST, right frontal aslant tract, forceps major, forceps minor, right inferior longitudinal fasciculus, and right vertical occipital fasciculus
**Qin 2021**	-	-	-	-	-	-	-	**Cortical thickness and subcortical volume comparison:** Considerable reduction of cortical thickness in the left superior temporal gyrus, left hippocampus, and left insula in the SG The negative relation between procalcitonin and cortical thickness in the SG **CBF comparison:** Compared with the NC, the MG exhibited a lower CBF value in the gray matter (the peak value in the left insula) Lower CBF value in SG in comparison with MG in left and right insula and bilateral superior medial frontal gyrus A positive relationship between the mean CBF value of the left insula in the SG and procalcitonin levels **White matter:** The MG as compared to NC displayed 17 tracts with alteration in three different parameters (FA: 2; length: 8; volume: 70) The SG in comparison with MG displayed 33 tracts alteration in three different measures (FA: 16; length: 5; Volume: 12)
**Huang/2021**	Lower in bilateral CR (anterior and superior), genu of the CC, and SLF L	-	-	-	-	-	-	**-**
**Benedetti/2021**	-	-	-	-	-	-	-	-No comparison with the control group -A seed in dorsal cingulate cortex identified with MVPA, showed negative correlation between IES-R score and connectivity to the medial prefrontal cortex and a positive correlation with connectivity to bilateral posterior cingulate cortex/precuneus in default mode network, superior temporal cortex in the sensorimotor network, and right middle frontal gyrus
**Yang/2021**	-	-	-	-	-	-	-	-Lower global efficiency (Eglob), longer shortest path length (Lp), and less nodal local efficiency in the superior occipital gyrus of cases
**Yildirim/2021**	-	-	-	-	-	-	-	**Olfactory Bulb MRI:** -Cases with COVID-19 OD had higher volumes in the Olfactory bulb compared to post-infectious OD -Olfactory sulcus depth showed no remarkable difference between the two groups - Overall, 58.1% of and 63.9% of post-infectious OD had deformed bulb morphology, without considerable difference between the two groups -Totally, 51.6% of COVID-19 related OD and 46.4% of those with post-infectious OD had increased olfactory bulb signals, without considerable difference between the two groups -A higher rate of olfactory nerve clumping was observed in COVID-19-related OD than post-infectious OD **Olfactory Tract DTI:** -Higher quantitative anisotropy value at orbitofrontal, entorhinal region, and orbitofrontal to entorhinal connections in COVID-19-related OD -Connection fibers between orbitofrontal and entorhinal regions are similarly affected on the left and right side in both groups, and asymmetry between connection fibers in the left and right side observed in 66.7% of COVID-19 OD and 67% of post-infectious OD -Connectogram maps demonstrate 42.3% dysconnectivity in post-infectious OD and 95.3% in COVID-19 OD on a visual scale **Olfactory fMRI:** -Orbitofrontal and entorhinal activity alone has no significant difference between groups -No orbitofrontal and entorhinal activity in 32.1% and 60.7% with COVID-19-related OD; and 29.5% and 45.3% with post-infectious OD -Robust trigeminosensory activity in COVID-19 related OD cases -Higher disorganized activity in post-infectious OD (23.7%) than COVID-19 (6.5%) related OD
**Silva/2020**	-	-	-	-	-	-	-	**Functional connectivity** -Higher global connectivity in patients -Inversion of connectivity direction in 30 pairs of ROIs in patients -The highest altered connection in the visuospatial network. No alteration in auditory network
**Lu/2020**	-	-	-	-	-	-	-	-Considerably higher mean global gray matter volumes -Markedly higher gray matter volume in the left Rolandic operculum area, bilateral olfactory cortices, bilateral insulas, bilateral hippocampi, right cingulate gyrus and left Heschl’s gyrus -No considerable differences in other regional gray matter volumes or any regional WM volumes
**Liang/2023**	-	-	-	-	-	-	-	-47 total MRI scans analyzed.9 abnormal scans identified (6 PCC, 3 controls).4 scans had more than age-related lesions (3 in PCC).2 scans with lacunar infarcts (both in PCC). 1 scan showed microhemorrhages (PCC).1 control scan had an old silent infarct and microhemorrhage. one control scan exhibited greater than age-related central atrophy. In DTI measures no extreme values found; all DTI scans included
**Qin/2024**								
**Chaganti/2024**								-PASC CI patient in comparison with controls had significant higher K-Trans and lower Glutamate/Glutamine values in frontal white matter and brainstem -No significant changes in PASC CI patients frontal Glutamate/Glutamine, but increase in brainstem Glutamate/Glutamine values -PASC CI had significant higher Myo-inositol values and decreased N-acetyl aspartate values in brainstem.
**Serrano del pueblo/2024**								-Long Covid patients showed significant cortical thinning in the posterior part of the left superior temporal gyrus, extending to the middle and inferior temporal gyri, compared to controls -Cortical thinning did not correlate with cognitive impairment levels -Rey memory test scores had a high correlation cluster in the temporal stem, important for memory
**Lipton/2024**			Decreased in Gyrus rectus and right middle orbitofrontal white matter of patients in comparison to controls which has minimal increase		Decrease in left orbitofrontal regions, right entorhinal gray matter and right uncinate fasciculus of patients compared to minimal increase of controls Less increase of ODI in the right inferior frontal gyrus in patients compared to controls			Left lateral anterior cingulate cortical thickness decreased in both case and controls significantly in cases ICVF of Gyrus rectus diminished in cases in comparison to controls which has minimal increase
**Petersen/2023**	-	-	-	-	-	-	-	-Covid group have increased peak width of skeletonized mean diffusivity (PSMD) and cortical thickness. However, none of the imaging markers, including these, demonstrated significant differences when compared to controls after adjusting for multiple comparisons -No significant difference of cortical thickness in vertex-wise analysis -Post-Covid subjects showed widespread increases in free water and MD across major white matter fiber bundles in all brain lobes, compared to more localized changes in other diffusion markers -Significant increases in free-water in 38.3% and decreases in 0.4% of the skeleton, along with elevations in tissue FA (FA_T_) in 3.3% of the skeleton, without any observed FAT reductions -A tract-of-interest analysis revealed significant increases in MD and free water across multiple white matter tracts, including association, commissural, and projection tracts -A supervised machine learning approach found that free water and MD were the strongest predictors, achieving median prediction accuracies of 80.21% and 79.38%, respectively, while cortical thickness scored 45.95%
**Arrigoni/2024**								-Significant atrophy of GM in Covid-CM compared to controls, and no GM atrophy in Covid-OD patients, but significant reduction in volume in specific individual GM regions after adjusting for age and sex differences.(localized rather than widespread GM changes) -Significant average cortical thinning in right hemisphere and posterior cingulate, isthmus cingulate, and parahippocampal cortex in both patients groups -Both patient groups exhibited a general rise in connectivity. alongside localized reductions, mainly in the left hemisphere. This increase was associated with reduced network modularity -Both groups exhibited increased clustering and local efficiency in certain regions, such as the right parahippocampal area, and decreased connectivity in others, like the insula. Covid-CM patients had strengthened connectivity in the right lingual region, while Covid-OD patients showed increased connectivity in areas like the left hippocampus and thalamus -The network-based statistic (NBS) approach identified significant connectivity alterations involving several brain regions, particularly the left insula, in both patient groups. These changes included decreased connectome density and varied effects on modularity and global efficiency between the two groups
**Fineschi/2024**								-No significant difference in he number of cortical infarcts, lacunar infarcts, white matter hyperintensities, global cerebral atrophy, or microbleeds between patients and controls -No significant difference in volume, cortical thickness or MR perfusion, in any of the anatomical regions comparing case and controls -No significant difference in gray matter VBM -No significant group difference of case and controls in white matter TBSS -Comparing cases to controls with resting fMRI, there were significant strong connectivity of the right middle frontal gyrus and significant weaker connectivity in the right inferior parietal lobule and the left fronto-parietal junction -Whole group showed significant positive correlation using Symptom Severity Scale specially in the right posterior temporoparietal junction and bilateral temporo-occipital junction and weaker correlations in the left frontobasal and left superior parietal areas. Also a minor negative correlation in the left parietal region
**Nelson/2024**								-No demyelinating evidence on patients MRI
**Sun/2025**								-No significant correlation between MDD and covid on gGM structure (two-way covariance analysis) -Infected HC and unaffected HC has no significant difference in cortical thickness, surface areas, and volumes. Also no significant difference between infected MDD and uninfected MDD -No significant difference of ALFF, ReHO and FC between infected HC and uninfected HC -Comparing infected MDD and uninfected MDD there were significant higher ALFF in left Amygdala, left Supplementary Motor Area, left Parahippocampal Gyrus, right Supplementary Motor Area, and lower AFF in left Angular Gyrus. But ReHo and FC has no significant difference -Investigating 20 major WM tracts, no significant correlation between MDD and covid-19 on ALFF, ReHo and FC -Uninfected HC group in comparison with infected HC showed increased FC between the left Anterior thalamic ra- diation and the left Superior longitudinal fasciculus, and between the Uncinate fasciculus and the left Superior longitudinal fasciculus Uninfected MDD comparing with infected MDD had increased ALFF value of the left Cingulum hippocampus and decreased ALFF of the right Uncinate fasciculus
**W.Churchill/2024**	No significant group differences, Post-hoc analyses within these clusters showed that clinical and demographic factors, such as symptom burden and time from symptom onset to MRI, did not significantly affect the results		No significant group differences, Post-hoc analyses within these clusters showed that clinical and demographic factors, such as symptom burden and time from symptom onset to MRI, did not significantly affect the results		No significant group differences, Post-hoc analyses within these clusters showed that clinical and demographic factors, such as symptom burden and time from symptom onset to MRI, did not significantly affect the results			-QA, RDI, and ISO has non significant trend -regional cerebral blood flow map had no sign of hypoperfusion, microbleeding, or vascular abnormalities -FLAIRE images and findings of MPRAGE images showed hyper intense lesions in subcortical and deep white matter of symptomatic post-covid patients
**Ibrahim/2024**								-No significant differences observed between the initial-visit COVID + and COVID- groups regarding DTI and DTI-DOME -significant group differences were observed in CDI values. The COVID- group showed higher log(CDI) in certain regions, with the most significant differences at lower b-values. The b = 1400 analysis revealed additional regions, such as the genu of the corpus callosum, while b = 2100 showed fewer significant effects but maintained significance in the superior corona radiata -Significant group differences were observed in the corona radiata and superior longitudinal fasciculus, especially in age-controlled analyses, highlighting widespread frontal effects -Conventional DTI, DT-DOME metrics, and NODDI parameters did not show significant group differences.-The COVID + group had higher log(CDI) in the cerebellum, with the largest differences at b = 2100, fewer differences at b = 700, and none at b = 1400 -Conventional DTI and DT-DOME metrics did not capture the group differences in the affected regions
**Teller/2023**								
**Balsak/2023**								apparent diffusion coefficient:Significant higher ADC in cases compare to controls in bulbus, pons and thalamus -Higher ADC values of putamen in group 3 compare to group 2 -Positive correlation between ADC of caudate nucleus and D dimer values
**Scardua‐Silva/2024**								No significant radiological changes on structural MRI There were no notable changes in the functional connectivity of the posterior cingulum cortex
**Boito /2023**								-Cc a parameter for structural orientational coherence within the voxel was lower in patients and it Cc differences mostly seen in occipital area -Most widespread differences were seen in FA, μFA, C_MD_ and RD, affecting large portion of the white matter -significant differences in several metrics (FA, MD,AD,RD,Cc, μFA, C_MD_)of patients and healthy controls (5.2% to 15.3%)
**Kausel/2024**								-Functional capacity, evaluated using the 6MWT, showed no differences between groups -Covid diagnosis and hospitalization did not affect brain activity related to decision-making or feedback. However, anosmia (loss of smell) was linked to reduced brain activity during decision-making in areas including the prefrontal cortex and temporoparietal regions -Covid diagnosis and hospitalization did not significantly affect cortical thickness -Anosmia correlated with cortical thinning in parietal areas - Affected white matter tracts included: the corticospinal tract, arcuate fasciculus, inferior fronto-occipital fasciculus, thalamus-parietal fasciculus, thalamus-occipital fasciculus, and posterior corpus callosum -Disruption in white matter integrity correlated with hospitalization and Covid diagnosis
**Deuter/2024**								-Significant difference in grey matter volume, white matter and CSF volume comparing patients with acute covid and recovered covid and also acute covid and healthy controls -Older age was significantly associated with decreased gray matter volume and increased CSF volume, with a trend towards decreased white matter volume -Recovered patients showed higher gray matter volumes in the cerebellum, fusiform gyrus, and hippocampus compared to acutely ill patients, while acutely ill patients had higher volume in the thalamus and basal ganglia -Compared to healthy controls, both acutely ill and recovered patients showed differences in the inferior frontal gyrus, insula, and basal ganglia. Cortical thickness was also reduced, especially in acutely ill patients -Comparing acute cases to controls, significant differences in ATR bilateral, CST bilateral, CBP left, IFO right, OR right, and FMI, acute cases generally showing lower volumes except for CBP left. comparing acute and recovered cases, notable differences in AF bilateral, ATR bilateral, CST bilateral, FAs bilateral, SLF3 bilateral, IFO right, OR right, STR right, FMA, and FMI, all with lower volumes in acute cases. comparing recovered cases to controls revealed no significant differences, except for STR right -Comparing acute cases and controls, significant differences were in SLF2 left, CBD left, and AF right, with acute cases exhibiting shorter tract lengths. comparing acute cases to recovered cases, notable differences were observed in SLF1 left, SLF3 left, and AC, with shorter tract lengths again seen in acute cases. no significant differences were detected between recovered cases and controls
**Trufanov et al./2024**								-Significant difference of subcortical structural difference in the right and left accessory nuclei -Only the dominant accessory nucleus correlated with the Head test, which assesses temporo-parietal-occipital and frontal function -Regression analysis revealed that the left nucleus accumbens size interacted with the default mode network in the right supramarginal gyrus - The left nucleus accumbens size had direct relationship with functional connectivity in the left occipital pole, fusiform gyrus, and cerebellar peduncle within the visual network -A direct connection between functional connectivity in the left frontal pole and middle frontal gyrus
**Planchuelo‐Gómez/ 2023**								-Covid patients in comparison with HC demonstrated lower GM volume in the bilateral pars orbitalis, and the right fusiform gyrus and frontal pole, and lower cortical thickness than HC in the right pars orbitalis - Covid patients in comparison with migraine patients had higher cortical thickness in the left paracentral cortex but lower subcortical volumes in the left accumbens and right thalamus. For chronic migraine patients, Covid showed lower cortical curvature values in certain areas and higher GM volume and thickness in various frontal and paracentral regions -Migraine patients (episodic and chronic) compare to HC displayed higher cortical curvature but lower cortical thickness, surface area, and GM volume across multiple brain regions. Notably, CM had lower GM volume than episodic migraine, while showing higher cortical thickness in specific areas like the inferior temporal gyrus
**Mishra/ 2024**								
**Lith/ 2024**					Voxel-based analyses: no significant differences in ODI and fCSF			**diffusion metrics:**At baseline, Covid group demonstrated elevated age- and sex-adjusted peak width of skeletonized mean diffusivity values. But with white matter hyperintensity volume correction it was not significant -no significant variations between Covid patients experiencing long Covid and those without long Covid. No significant differences between ICU patients and non-ICU patients -Reduced NDI values in the right anterior thalamic radiation, forceps minor, and right inferior fronto-occipital fasciculus **MRI:** After three months, orientation dispersion index values increased in several brain regions compared to baseline (parts of the corticospinal tract, cingulum, and other fasciculi) -This increase remained significant even when accounting for changes in white matter hyperintensity volume

Diffusion tensor imaging (DTI), Neurite orientation dispersion and density imaging (NODDI), Functional magnetic resonance imaging (fMRI), Tract-based spatial statistics (TBSS), Voxel-based morphometry (VBM), Fractional anisotropy (FA), Mean diffusivity (MD), Axial diffusivity (AD), Radial diffusivity (RD), Volume fraction of intracellular compartment (Vic), Volume fraction of extracellular compartment (Vec), Volume fraction of cerebrospinal fluid (VCSF), Volume fraction of isotropic diffusion compartment (Viso), Orientation dispersion index (ODI), Fiber density (FD), Apparent fiber density (AFD), White matter (WM), Corpus callosum (CC), Corona radiata (CR), Free water (FW), Superior longitudinal fasciculus (SLF), Inferior fronto-occipital fasciculus (IFOF), resting state-Functional connectivity (rs-FC), Cerebral blood flow (CBF), Mild group (MG), Severe group (SG), Normal control (NC), Healthy control (HC), Intensive care unit (ICU), C-reactive protein (CRP), Impact of Event Scale- Revised (IES-R), PTSD checklist-civilian version (PCL-C), Posttraumatic Stress Disorder Self-Rating Scale (PTSD-SS), Generalized Anxiety Disorder Screener (GAD-7), Olfactory dysfunction (OD), participants with post-COVID-19 conditions (PCC), post-acute sequelae of SARS-COV-2 infection (PASC),cognitive impairment (CI), body of the corpus callosum (BCC), splenium of the corpus callosum (SCC), right posterior limb of the internal capsule (PLIC), left superior corona radiata (SCR), bilateral posterior corona radiata (PCR), bilateral posterior thalamic radiation (PTR), bilateral anterior thalamic radiation (ATR), bilateral corticospinal tract (CST), forceps major (fmajor), right inferior longitudinal fasciculus (ILF), right inferior fronto-occipital fasciculus (IFOF), bilateral superior cerebellar peduncle (SCP), right cerebral peduncle (CP),orientation dispersion index (ODI), intracellular volume fraction (ICVF),Covid patients with olfactory (Covid-OD), cognitive impairment (Covid-CM),amplitude of low-frequency fluctuations (ALFF), major depression disorder (MDD), magnetization prepared rapid gradient echo single-shot echo- planar imaging sequence (MPRAGE), correlated diffusion imaging (CDI),apparent diffusion coefficient (ADC),orientational coherence (Cc) and variance in compartment’s size (CMD),,Minute Walk Test (MWT),Episodic migraine(EM), Chronic migraine (CM),(AC), Arcuate Fasciculus (AF), Acoustic Radiation (AR), Anterior Thalamic Radiation (ATR), dorsal/ peri-genual and temporal Cingulum subsection (CBD, CBP, CBT), Corticospinal Tract (CST), Frontal Aslant (FAs), Forceps Major and Minor (FMA, FMI), Fornix (FX), Inferior Longitudinal Fasciculus (ILF), Inferior Fronto-Occipital Fasciculus (IFO), Middle Cerebellar Peduncle (MCP), Middle Longitudinal Fasciculus (MdLF), Optic Radiation (OR), Superior Thalamic Radiation (STR), Superior Longitudinal Fasciculus 2 (SLF2), Uncinate Fasciculus (UF) and Vertical Occipital Fasciculus (VOF)

Abnormal diffusion parameter in the corpus callosum (CC) were mentioned in seventeen studies (Huang et al. 2022; Huang et al. 2023; Silva et al. 2021; Yang et al. 2021; Campabadal et al. 2023; Díez-Cirarda et al. 2022; Paolini et al. 2023; Qin et al. 2024; Chaganti et al. 2024; Serrano et al. 2024; Arrigoni et al. 2024; Churchill et al. 2024; Ibrahim et al. 2024; Trufanov et al. 2025; Planchuelo-Gómez et al. 2023). Three studies (Silva et al. 2021; Chaganti et al. 2024; Trufanov et al. 2025) noted an increased FA in this tract in the left tapetum (Trufanov et al. 2025) and genu (Silva et al. 2021). Silva and colleagues also found a higher MD and AD in the splenium of CC (Silva et al. 2021). On the contrary, Diez et al. (Díez-Cirarda et al. 2022) reported reduced MD and AD in the CC. A similar finding was reported by Churchill et al. (Churchill et al. 2024) in the splenium, and Chaganti et al. (Chaganti et al. 2024). Four studies (Yang et al. 2021; Qin et al. 2024; Serrano et al. 2024; Planchuelo-Gómez et al. 2023) noted reduced FA in CC, of which Qin et al. (Qin et al. 2024) found lower AD and higher RD, and Yang and colleagues (Yang et al. 2021) also reported an elevated MD and RD in the CC. Likewise, other three studies (Campabadal et al. 2023; Paolini et al. 2023; Arrigoni et al. 2024) showed that MD was increased in multiple sections of CC. Nine studies (Huang et al. 2022; Huang et al. 2023; Yang et al. 2021; Campabadal et al. 2023; Paolini et al. 2023; Qin et al. 2024; Arrigoni et al. 2024; Churchill et al. 2024; Planchuelo-Gómez et al. 2023) focusing on corona radiata (CR) consistently noted abnormal diffusion metrics, i.e. higher values of MD and RD (Yang et al. 2021; Campabadal et al. 2023; Paolini et al. 2023; Qin et al. 2024; Arrigoni et al. 2024). The same pattern was observed in the left cingulum (Yang et al. 2021; Paolini et al. 2023). On the other hand, lower FA was observed by two studies (Qin et al. 2024; Planchuelo-Gómez et al. 2023) in CR.

Longitudinal fasciculi (LF) were amongst the tracts showing alteration in COVID-19 with more consistent results in seventeen studies (Bispo et al. 2022; Huang et al. 2022; Huang et al. 2023; Silva et al. 2021; Tian et al. 2022; Yang et al. 2021; Díez-Cirarda et al. 2022; Paolini et al. 2023; Pelizzari et al. 2022; Liang et al. 2023; Qin et al. 2024; 2024; Sun et al. 2025; Ibrahim et al. 2024; Deuter et al. 2024; Trufanov et al. 2025; Planchuelo-Gómez et al. 2023). Six studies mentioned lower FA in the superior LF (SLF) (Yang et al. 2021; Pelizzari et al. 2022; Serrano et al. 2024; Sun et al. 2025; Deuter et al. 2024), inferior LF (ILF) (Qin et al. 2024; Serrano et al. 2024) and bilateral middle LF (MLF) (Deuter et al. 2024). In contrast, three studies reported an increased FA in ILF (Silva et al. 2021; Trufanov et al. 2025) and SLF (Liang et al. 2023). Only two studies (Díez-Cirarda et al. 2022; Deuter et al. 2024) noted MD changes in these tracts, of which Diez et al. (Díez-Cirarda et al. 2022) observed a decrease in MLF, while Deuter et al. (Deuter et al. 2024) showed an increase in bilateral MLF and left SLF. Studies evaluating AD and RD in these tracts (Bispo et al. 2022; Díez-Cirarda et al. 2022; Paolini et al. 2023; Pelizzari et al. 2022; Liang et al. 2023; Qin et al. 2024; Planchuelo-Gómez et al. 2023) consistently reported a lower AD and a higher RD, except for Diez et al. (Díez-Cirarda et al. 2022) and Liang et al. (Liang et al. 2023), of which the latter reported lower RD in the right SLF I. Besides, Bispo and colleagues stated that the FD and AFD total values were lower in the SLF of their cases (Bispo et al. 2022). Incongruent results on uncinate fasciculi (UF) were reported in thirteen studies (Bispo et al. 2022; Lu et al. 2020; Silva et al. 2021; Tian et al. 2022; Campabadal et al. 2023; Díez-Cirarda et al. 2022; Paolini et al. 2023; Serrano et al. 2024; Lipton et al. 2024; Arrigoni et al. 2024; Deuter et al. 2024; Trufanov et al. 2025; Mishra et al. 2024). Three studies found an increased RD in these tracts (Campabadal et al. 2023; Paolini et al. 2023; Mishra et al. 2024) alongside an increased MD associated with COVID-19 (Campabadal et al. 2023; Paolini et al. 2023; Deuter et al. 2024). In contrast, Diez et al. (Díez-Cirarda et al. 2022) and Silva et al. (2021) showed that MD was significantly decreased in COVID-19. Silva and colleagues (2021) reported an increased FA in UF, and Trufanov et al. mentioned this finding only on the left side (Trufanov et al. 2025). Inconsistent with these findings, three studies showed lower FA in UF (Serrano et al. 2024; Arrigoni et al. 2024; Mishra et al. 2024).

**Fig. 2:**
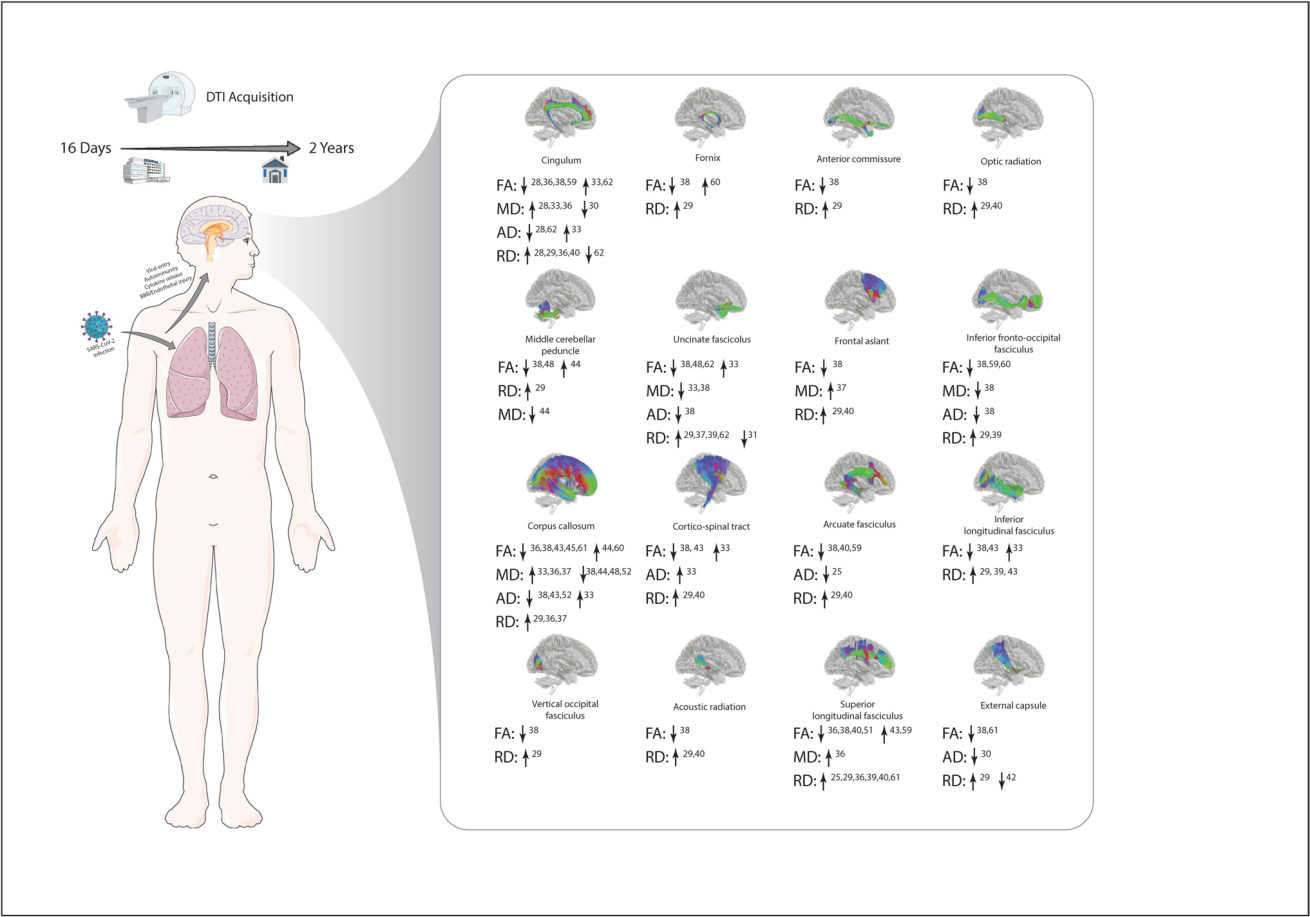
Overview of the changes in DTI metrics in COVID-19 patients. The imaging timeline varied from 16 days to 2 years in included studies. Each upward arrow means an increase in diffusion parameters and each downward arrow mean a decrease in diffusion parameters in one study. The fiber tract visualization was adopted from the Human Connectome Project population-based atlas (available at https://brain.labsolver.org/hcp_trk_atlas.html). DTI: diffusion tensor imaging, FA: fractional anisotropy, MD: mean diffusivity, RD: radial diffusivity, AD: axial diffusivity

Ten studies mentioned alterations in the fronto-occipital fasciculus (FOF) (Bispo et al. 2022; Lu et al. 2020; Yang et al. 2021; Díez-Cirarda et al. 2022; Paolini et al. 2023; Pelizzari et al. 2022; Liang et al. 2023; Kausel et al. 2024; Trufanov et al. 2025; Lith et al. 2024). Yang et al. (Yang et al. 2021) and Qin et al. (Qin et al. 2024) mentioned a reduced FA in the left superior FOF (SFOF) and right inferior FOF (IFOF), respectively. In contrast, Trufanov and colleagues (Trufanov et al. 2025) showed that FA was increased in the left IFOF. Increased RD was reported by Pelizzari et al. (Pelizzari et al. 2022) and Paolini et al. (Paolini et al. 2023) in the IFOF, while only the latter reported increased MD. In contrast, Diez and colleagues (Díez-Cirarda et al. 2022) and Lu et al. (Lu et al. 2020) found MD and AD to be decreased in the FOF and right SFOF, respectively. A decreased AD and higher RD was also noted by Qin et al. (Qin et al. 2024). Table 2 demonstrates detailed information on white matter tracts differences and Fig. 2 provides a brief illustration of the main findings.

#### Mental status and correlations between DTI results and cognitive findings

Of the included studies, eleven reported significant difference between COVID-19 and controls in neurocognitive functions in the study groups and correlations between cognitive findings and DTI measurements (Bispo et al. 2022; Benedetti et al. 2021; Huang et al. 2022; Huang et al. 2023; Silva et al. 2021; Yang et al. 2021; Díez-Cirarda et al. 2022; 2024; Petersen et al. 2023; Arrigoni et al. 2024; Lith et al. 2024), as shown in Table 3. Benedetti and colleagues reported that although the systemic immune-inflammation index (SII) score on admission had a positive correlation with Zung Self-rating Depression Scale (ZSDS), its decrease during treatment was associated with higher ZSDS scores (Benedetti et al. 2021). In the study by Silva et al. (2021), depressive symptoms in the participants were correlated with The Chalder Fatigue Scale (CFQ-11) scores. Furthermore, Bispo and colleagues (Bispo et al. 2022) reported higher CFQ-11 scores, indicating higher mental and physical fatigue, in the COVID-19 group. Besides, tract average FA and RD in the CC, ILF and SLF, arcuate fasciculus, cingulum, and fornix were associated with processing speed. They also mentioned that tract-average FA and AD in CST and CR were negatively associated with CFQ-11 scores (Bispo et al. 2022). Moreover, Serrano del Pueblo and colleagues (2024) stated that lower FA in various WM clusters including cingulum bundle, temporal stem, and corpus callosum was significantly correlated with poorer cognitive outcomes; such as lower overall cognitive level (OCL) and object recognition scores, and orientation in time and place. On the other hand, Petersen et al. (Petersen et al. 2023) found MD to have positive correlation with Trail Making Tests (TMT), and negative correlation with verbal fluency and word list recall. Additionally, van Lith and colleagues (Lith et al. 2024) noted an association between lower NDI and lower post-COVID-19 functional status scale.

**Table 3:** The overview of the correlation between diffusion parameters and cognitive findings

Study	Mental status in case vs. control group	Investigated correlation of diffusion metrics with cognitive function: Significant findings	Association between diffusion metrics and other parameters
Campabadal 2022	-	-	-No significant relationship between the olfactory system GM volume and the UPSIT -The UPSIT was negatively associated with both MD and RD values for the whole sample, in the anterior thalamic radiation, orbitofrontal WM tracts, the genu of the corpus callosum, and the forceps minor
Bispo 2022	-The COVID-19 + group scored higher on the total CFQ-11 scale, physical fatigue, and mental fatigue -The COVID-19 + group did not differ from COVID-19-in MoCA global score and CANTAB neurocognitive performance	- Tract-average FW values in the right fornix were correlated with visual memory measures—PALTEA and PALFAMS in the COV + group. A correlation was identified between right fornix microstructural measures and visual memory for RDt, MDt, and ADt. Tract-average FAt, RDt, and FW values associated with processing speed (single-choice movement and reaction time—RTISMDMT and RTISMDRT, and five-choice reaction time, RTIFMDRT) In the COV + group. Tract-average d-MRI measures were associated with these processing speed measures in the cingulum, corpus callosum, arcuate fasciculus, inferior & superior longitudinal fasciculus, and fornix. MoCA and WM measures were not correlated. There is no correlation between D-MRI metrics, MoCA, and CANTAB subtests in the COV- group	-In the COV + group, tract-average FD values in the right & left corticospinal tract, left & right corona radiate, middle cerebellar peduncle, and posterior mid body of the corpus callosum were negatively correlated with total CFQ-11 score. The tract average FAt measurements were negatively associated with the total CFQ-11 score in the corticospinal tract, corona radiata, and corpus callosum. The tract-average ADt were negatively associated with the total CFQ-11 score measurements in the corticospinal tract, corona radiata, and superior longitudinal right fasciculus -In the COV + group, tract-average FD values in the corticospinal tract, corpus callosum, corona radiata, and the middle cerebellar peduncle were negatively associated with physical fatigue. The FD values and mental fatigue were not correlated. The d-MRI metrics and CFQ-11 scores (mental, physical, and total) were not correlated in the COV- group
Huang 2023	Comparing PT2 and HC2: significant different in LM-A and LM-B. In acute stage negative association between LM-A and CRP, ESR -In acute stage positive correlation between Viso and ESR and negative correlation between LM-A and SII in PT2 group -Negative correlation LM-A and Viso (partial correlation within all PT2 and HC2) -After comparison corrections the only correlation in recovered covid patients: between Viso and ESR, also LM-A and SII	-	-
Diez-Cirarda 2022	-Higher cognitive dysfunction in hospitalized patients (in language, memory, working memory and attention, visuospatial ability, processing speed) -cognitive dysfunction significantly related to GM atrophy (mainly processing speed working memory and attention) -lower GM volume associated with poor cognitive performance (memory and visuospatial)	-	-MD was higher in hospitalized patients compare to non-hospitalized
Paolini 2023	-	FA values mild decrease in cognitive complainers	-
Pelizzari/2022	-	-	-
Rau 2022	MoCA score; 22.4 ± 4.9	Significant negative correlation between MoCA test and V-CSF	-No significant correlation between V-CSF and olfactory performance or IL-6 values. -A significant correlation displayed between cerebral glucose metabolism and V-CSF
Tian 2022	-	-	-
Qin 2021	-	-	-A positive association between FA and IL-6 on the left middle longitudinal fasciculus -A significant negative association between volumes of the right anterior thalamic radiation and right inferior longitudinal fasciculus with procalcitonin level in MG - A notable positive relation between volumes of the left CST and right optic radiation with IL-6
Huang 2021	For cognitive function evaluation, either between the recovered COVID patients and the healthy group or the ICU and non-ICU group there were no significant difference in the Wechsler Intelligence scale (5 tests for cognitive function: LM_A,LM_B,DSST,Knowledge subscale of Wechsler Intelligence scale, FDS, BDS, WFT)	-	-V_ic_ of bilateral corona radiata (anterior and superior part), genu of the corpus callosum, and left superior longitudinal fasciculus has a negative correlation with length of hospitalization and a positive correlation with days of follow-up
Bendetti 2021	-Post-acute psychopathology in the emergency department can be predicted by inflammatory markers -SII at hospital admission has a positive correlation with ZSDS. SII decrease during treatment has negative correlation with ZSDS. A high correlation between SII at hospital discharge and ZSDS. -SII at hospital discharge has a positive correlation with IES-R scores -Delta SII has a negative correlation with IES-R -CRP and ZSDS at hospital are also positively correlated, but CRP does not show a high correlation with IES-R	-Negative correlation between the severity of psychopathology as measured by ZSDS, BDI, and IES-R and gray matter volume in VBM analysis. (in large clusters in bilateral ACC, encompassing Brodmann areas (BA) 24 and 32)) -Association between BDI and IES-R with gray matter volumes. (In bilateral insular cortex) -Negative correlation between IES-R and AD in several white matter tracts in both hemispheres (superior and posterior corona radiata and superior longitudinal fasciculus and Inferior longitudinal fasciculus, external capsule, anterior thalamic radiation) -Negative correlation between BDI and AD in a small cluster (left superior corona radiata, superior longitudinal fasciculus, and posterior corona radiata)	-Inflammatory markers predict DTI measures of white matter microstructure but has no effect on VBM -Negative association between SII and AD, MD -Negative association between CRP and AD -Multivariate pattern analysis (MVPA) displayed SII and rs-FC association in 5 seeds: left dorsolateral prefrontal cortex (salience network), inferior parietal sulcus (dorsal attentional network), cerebellum, right parietal lobe, and precentral gyrus (dorsal attentional network) -Inflammatory markers affect resting-state FC -Severity of inflammation showed a negative association with rs-FC and a positive association with FC -Negative correlation between CRP and rs-FC in between right caudate and two clusters in the right temporal cortex in the language network
Yang2021	-Mental health scores: PHQ-9, GAD-7, HAMD, HAMA, PCL-C, PTSD-SS -Purine pathway which is related to the mental health alteration disturbed	-Remarkably positive correlation between FA and the PCL-C, PTSD-SS, and GAD-7 scores. (in the left superior corona radiate, the body of corpus callosum, and left posterior thalamic radiation) -Considerably negative correlation between the RD and PCL-C, PTSD-SS, and GAD-7 scores. (in the left superior corona radiate, the body of corpus callosum, left external capsule, left anterior limb of the internal capsule, and posterior thalamic radiation) -The MD had a significant negative correlation with the PCL-C in the corpus callosum -PCL-C had a remarkable positive correlation with Eglob and a negative correlation with Lp	-Significant correlation between elevated AA and FA decrease, MD increase, and RD increase in some brain regions (left cingulum, left anterior corona radiate, and left internal capsule) -Positive correlation between FA value in the left anterior corona radiate and serotonin level -Relationship between white matter abnormalities of the frontal limbic system and symptoms of PTSD and anxiety -Differential metabolites displayed considerable association with the level of Eglob, Lp, FA, RD, or MD
Yildirim 2021	-	-	-
Silva 2020	BDI-II > 13 in 18%, BAI > 10 in 29% of subjects Symptoms of depression correlated with CFQ-11 score Abnormal performance in phonological fluency (33%), TMT-A (30%), TMT-B (40%)	-Mild association between right ILF-FA and TRAIL-B -No association between ILF and Phonological fluency, or the logical memory scores (immediate and delayed recall) and diffusivities in the parahippocampal cinguli	-
Lu 2020	-	-	-No statistically significant differences were found in the indices of interest between patients with and without neurological symptoms during acute stage, patients with and without neurological symptoms at the follow-up point, or patients classified as non-severe and severe -No significant differences between patients with (n = 2) and without (n = 58) smell loss in regional GMVs and MD of GM -Significantly positive relationship between vision changes during the acute stage, and MD of GM in the right thalamus, AD of WM in right EC hippocampus, and GMVs in right cingulate revealed a negative correlation with smell loss -Global GMV, MD-GM score had a negative correlation with LDH and a positive correlation with the right cingulate gyrus of GMV -The FA-WM score displayed a negative correlation with tremor and a positive correlation to the MD-WM score -Global GMV and regional GMV in the left Rolandic operculum, right cingulate, bilateral hippocampus, and the left Heschl’s gyrus, also global MD value of WM displayed all negatively related to memory loss -MD of GM in cingulate gyri was positively related to fatigue and numbness -MD of GM in the right precuneus displayed a positive correlation with numbness
Liang/2023	-Similar performance in cognitive assessments between groups. PCC group had higher perceived stress and depression, anxiety levels (p = 0.001), greater fatigue and pain interference (p < 0.001). Also poorer global mental health in PCC participants (p < 0.001)	-	-Higher FA in bilateral sagittal stratum predicted higher fatigue T-scores -Higher MD in left amygdala predicted greater perceived stress -Women with higher MD reported more fatigue and anxiety -No DTI measures correlated with time since Covid diagnosis
Qin/2024	-	-	**Right IFOF:** Significant negative correlation between FA value and Pittsburgh Sleep Quality Index (PSQI) score, sub-PSQI (sleep quality, use of sleeping medication) Significant negative correlation between AD value and PSQI score and use of seeping medication Significant positive correlation between RD value and sleep quality **Left CST:** significant positive correlation between FA and PSQI and sub-PSQI scores (habitual sleep efficiency, daytime dysfunction, sleep quality and sleep disturbances) Significant negative correlation between RD and PSQI and sub-PSQI scores (sleep efficiency, sleep quality, the use of sleeping medication and daytime dysfunction) Significant positive correlation between IL-1β and PSQI, sub-PSQI scores (sleep quality, sleep latency, and sleep disturbances) After 3 month, no changes in ROI analysis of left corticospinal tract, but significant changes of FA and RD in right inferior fronto-occipital
Chaganti/2024	No association among K-Trans and neurocognitive scores in brainstem		Negative correlation among frontal white matter K-Trans and FA
Serrano del pueblo/2024	-During acute Covid infection, neurological symptoms like memory failures and dizziness were associated with poorer attention and episodic memory in patients -Common long Covid symptoms included memory issues, fatigue, headaches, and anxiety. While some symptoms like anosmia improved over time, others like fatigue and headaches persisted -Only insomnia among long Covid symptoms was significantly linked to episodic memory deficits. Anxiety and depression were more prevalent in long Covid patients but did not correlate with cognitive impairment -Overall, patients showed significant cognitive impairments, with 27% scoring below the 24th percentile in overall cognitive level (OCL). Episodic memory was particularly affected, with 48% of patients performing poorly in this domain -Patients had notable deficits in episodic memory, attention, verbal fluency, and processing speed. However, language, visuospatial perception, and premorbid IQ were relatively preserved -While some executive functions like problem-solving and divided attention were impaired, higher-order executive tasks were generally preserved in most patients, except for those with severe cognitive impairment -Patients with more severe cognitive impairment (below the 24th percentile) showed poorer performance in executive functions and episodic memory compared to those with higher cognitive scores. Even in the better-performing subgroup, episodic memory remained impaired	-Lower FA values in white matter were associated with poorer cognitive outcomes, including verbal fluency, attention, and episodic memory. Specific cognitive domains correlated with FA changes in distinct white matter regions, such as the temporal stem for memory and the cingulum bundle for object recognition -No correlations between FA and cognitive outcomes in Covid recovered patients -Significant correlations between FA and OCL verbal fluency, attention, and episodic memory (verbal and visual domains) in the patient group -Lower OCL was significantly associated with lower FA values in various white matter regions, including the frontal lobe, thalamus, fornix, STG white matter, temporal stem, cingulum bundle, internal capsule, parietal, and occipital white matter -Lower FA scores correlated significantly with lower measures of verbal fluency, attention, and memory (delayed visual free recall, object recognition, time, and spatial orientation) -There was substantial overlap in white matter areas associated with outcomes in different cognitive domains, with some specificity: Attention scores correlated with white matter of the STG -Object recognition scores correlated with FA in the cingulum bundle, splenium of the corpus callosum, right temporal stem, and posterior optic radiations -Orientation in time and place correlated primarily with FA in the internal capsule and the splenium of the corpus callosum	
Lipton/2024	-Controls showed improvement in most cognitive tasks, which is expected due to practice effects. (Identification task , Groton Maze Chase Task, International Shopping List immediate recall, One-Back task, and Two-Back task) -showed a decline in performance in Identification task, Groton Maze Chase Task, One-Back task and Two-Back task -Despite these differences in direction, the overall change in cognitive performance between patients and controls was not statistically significant	-	-
Petersen/2023	Post-SARS-CoV-2 individuals initially demonstrated nominally better performances in Verbal Fluency, Mini-Mental State Examination, and clock drawing test when compared to matched controls. However, after applying the Bonferroni correction for multiple comparisons, no significant differences were observed in any neuropsychological test scores, including those related to executive functioning, memory, psychosocial, and neurological symptom burden	-MD showed positive associations with Trail Making Tests (TMT-A and TMT-B), and negative associations with Verbal Fluency and WLR. These correlations were significant only in the post-Covid cases	-Free water was positively associated with slower performance on the Trail Making Tests (TMT-A and TMT-B) and negatively associated with verbal fluency and Word List Recall in the entire sample. These associations were stronger in post-Covid cases -Both free water and MD increased with age, with stronger effects observed in post-Covid patients
Arrigoni/2024		-Significant FA change in WM of covid-CM at global level.(after adjusting for age, sex and BrainsegVol it was not significant) -Decreased FA of WM in tracts within the medulla in COVID-CM patients -Significant MD increase in overall GM in both Covid-CM and Covid-OD groups -Significant WM MD increase of Covid-CM compare to controls -Tractometry and MD regional analysis on major WM bundles showed a significant increase in MD within the forceps minor section of the corpus callosum in Covid-CM patients	-Decreased FA of WM in the middle cerebellar peduncle in the Covid-OD group -Significant FA decrease in the right uncinate fasciculus (UF) in Covid-OD patients -Significant MD increase in overall GM in both Covid-CM and Covid-OD groups
Fineschi/2024	-Significant positive correlation between BRANS (Repeatable Battery for the Assessment of Neuropsychological Status Update) total index score of gray matter volume in the right superior/middle-temporal gyrus		
Nelson/2024	-No significant difference of fluid cognition between long covid and normal recovered -After adjusting for crystallized cognition and education, no differences were found in fluid cognition test scores		Post hoc analysis revealed that the long Covid group scored lower on the Picture Sequence Memory task compared to the normal recovered group, particularly in terms of educational attainment
Sun/2025	No significant difference in age of onset, total duration, duration of current episode, Hamilton Depression Rating Scale-24 items total scores, Hamilton Anxiety Rating Scale total scores, and Snaith-Hamilton Pleasure Scale total scores between iMDD and uiMDD between infected MDD and uninfected MDD	-	-
W.Churchill/2024	**SocSat** (social satisfaction): Both Covid-19 and control groups had lower scores than the normative mean **WelBei** (well-being): Both groups also had lower scores than the normative mean **NegAff** (negative affect): The Covid-19 group had significantly higher scores than the normative mean, while the control group did not (Despite these differences from the normative mean, there were no significant differences in average summary scores between the Covid-19 and control groups.) The Covid-19 group had higher composite scores for SocSat and NegAff, indicating these factors were more prominent Positive correlations were found between NIH emotional composite scores and viral symptoms, such as gastrointestinal issues, headache, and fatigue (significant at FDR 0.05), as well as cough, shortness of breath, and total symptoms (significant at uncorrected p < 0.05)	No significant associations between composite score and DTI measurements. However, ODI showed significant positive associations in the right posterior limb of the internal capsule, and VISO exhibited significant negative associations in the superior longitudinal fasciculus	-
Ibrahim/2024	-	-	-
Balsak/2023	-	-	Negative correlation between FA of mentioned areas and plasma LDH
Scardua‐Silva/2024	Prevalent post-acute symptoms are: memory difficulties(36.1%), fatigue (30.9%), headache (28.9%), and concentration difficulties (20.6%). Fatigue mostly presented with other symptoms	No significant association between neuropsychological scores and neuroimaging features (including DTI and RS‐fMRI): linear regression analyses showed no significant relationships between the functional connectivity maps and the Epworth Sleepiness Scale and Chalder Fatigue Questionnaire-11 (brain connectivity maps and scores for fatigue or sleepiness.) Individuals with mild infections showed persistent cognitive impairments and subtle abnormalities in white matter without anxiety or depression symptoms	-
Boito et al./2023		-	-
Kausel/2024	- The frequency of cognitive symptoms was not linked to clinical factors like anosmia or hospitalization - The ACE-III cognitive evaluation showed an average score of 92, with no significant differences between groups - The IFS-Ch frontal screening evaluation showed an average score of 21.8, with no significant differences between groups - The PHQ-9 depression screening showed no significant differences between groups - The GAD-7 anxiety screening showed no significant differences between groups	Covid-19 diagnosis, anosmia, and hospitalization were not associated with significant differences in cognitive or psychological assessment scores	
Deuter/2024	The Kruskal–Wallis test showed significant differences among the study groups in cognitive tests, including Logical Memory I, Trail Making Test B, Regensburg Word Fluency Test (multiple categories), and the Digit Symbol Coding Test		
Trufanov et al./2024	-	-	-
Planchuelo‐Gómez/2023	-	-	-A significant negative correlation was observed between mean AD values and disease duration in Covid patients in the left posterior corona radiata. But no significant correlation for mean FA, RD or MD -Significant negative correlations between disease duration and GM volume in the pars opercularis, superior frontal gyrus, and insula -Negative correlation was found between disease duration and surface area in the insula -No significant correlations regarding headache frequency in both migraine group -No significant correlation between diffusion measurements in white matter and total migraine duration -No statistically significant correlations for chronic migraine patients
Mishra/2024	-	-	Significant FA decrease and increase RD changes in left UF of controls comparing to either hospitalized or non-hospitalized patients Significant FA reduction in right cingulum cingulate when comparing healthy group to either hospitalized or non-hospitalized patients Increased FA in right cingulum hippocampus of patients comparing to health controls. (With significantly difference between or non-hospitalized patients and HCs) Finally in all comparisons there were not significant difference between HP and or non-hospitalized patients
Lith/2024	-Compared to controls, Covid patients exhibited poorer functional outcomes both 3 and 12 months following discharge -Patients exhibited increased depression symptoms (p = 0.008), scored lower on cognitive functions as assessed by TICS-M at 12 months, and had reduced scores on the VAS scale (p = 0.006) -Excluding a reduction in the occurrence of long Covid, patients showed no differences in functional and cognitive outcomes between the 3-month and 12-month follow-up periods	-Despite adjusting for WMH volumes (p = 0.018), the regions with lower NDI values, which were distinct between patients and controls at baseline, continued to be significantly linked to lower PCFS scale scores (functional outcome) at the 12-month follow-up	

MoCA: Montreal Cognitive Assessment, UPSIT: University of Pennsylvania Smell Identification Test, COV: Covid-19, CANTAB: Cambridge Neuropsychological Test Automated Battery, PALTEA: Paired associates learning total errors adjusted, PALFAMS: Paired associates learning first attempt memory score, FAt: Tissue fractional anisotropy, RDt: Tissue radial diffusivity, ADt: Tissue axial diffusivity, MDt: Tissue mean diffusivity D-MRI: diffusion-weighted MRI, WM: White membrane, PT2:recovered patients at two years after discharge, HC2: Healthy control group in 2022, V-CSF: free water/CSF fraction, CSF: Cerebrospinal fluid, IL-6:interleukin-6, ICU: intensive care unit, LM-A: logical memory immediately, LM-B: logical memory after 30 min, DSST: digit symbol substitution test, FDS: forward digit span, BDS: backward digit span,WFT: word fluency test,Vic: volume fraction of intracellular water, SII: Systemic immune-inflammation index, ZSDS: Zung Self-Rating Depression Scale, IES-R: Impact of Event Scale-revised, ESR: Erythrocyte sedimentation rate, CRP:C-reactive protein,BDI: Beck Depression Inventory,ACC: anterior cingulate,VBM: voxel-based morphometry, AD: axial diffusivity, MVPA: multivariate pattern analysis,DMN: default mode network,MD: mean diffusivity, RD: radial diffusivity, PHQ-9: 9-Patient Health Questionnaire, GAD-7:Patient Health Questionnaire, HAMD: Hamilton Depression Rating Scale, HAMA: Hamilton Anxiety Rating Scale, PCL-C:PTSD checklist-civilian version, PTSD-SS: Posttraumatic Stress Disorder Self-Rating Scale, Eglob: global efficiency, Lp: shortest path length,BAI: Beck Anxiety Inventory, CFQ-11: Chalder Fatigue Questionnaire, TMT-A: Trail Making Test, ILF: inferior longitudinal fasciculus, post-COVID-19 conditions: PCC:participants with post-COVID-19 conditions, OCL:overall cognitive level, WRL: word recall list, Covid-CM:Covid patients with cognitive impairment, Covid-OD: Covid patients with olfactory disease, MDD: major depressive disorder, ACE-III: Addenbrooke’s Cognitive Examination, (IFS-Ch: Ineco Frontal Screening, PHQ-9: Patient Health Questionnaire-9, GAD-7: Generalized Anxiety Disorder 7-item, VAS: Visual Analogue Scale, TICS-M: Telephone Interview for Cognitive Status, WMH: white matter Hyperintensities, NDI: lower neurite density index, PCFS: Post-COVID-19 Functional Status scale

Assessing the participants with Montreal cognitive assessment (MoCA), Bispo et al. found no correlation between MoCA and WM measures (Bispo et al. 2022). Benedetti et al. (Benedetti et al. 2021) demonstrated negative correlations between Impact of Event Scale—Revised (IES-R) and Beck’s Depression Inventory (BDI) scores, and AD in the SLF and posterior CR on both sides. Yang and colleagues (Yang et al. 2021) stated that there was a positive correlation between scores on the Post-traumatic stress disorder (PTSD) checklist – civilian version (PCL-C), PTSD self-reporting scale, and the Generalized anxiety disorder (GAD) screener and the FA in the left superior CR, the body of the CC, and the left posterior thalamic radiation. In addition to the tracts mentioned above, in the left EC, left anterior limb of the internal capsule, and right posterior thalamic radiation, RD was negatively correlated with the same scores, whereas MD in the CC had a negative correlation only with PCL-C. Also, PCL-C was positively and negatively correlated with global efficiency and the shortest path length (Yang et al. 2021). Serrano del Pueblo et al. (2024) mentioned significant cognitive impairment in 27% scoring lower than 24th percentile in OCL, particularly episodic memory, although language, visuospatial perception and premorbid IQ was preserved. Additionally, Deuter et al. (Deuter et al. 2024) observed significant difference between study groups in logical memory, TMTs, Regensburg Fluency Test, and the Digit Symbol Coding Test. Besides, van Lith and colleagues (Lith et al. 2024) found decreased cognitive function in patients after 12 months, assessed by the telephone interview for cognitive status (TICS-M) test.

## Discussion

We systematically reviewed the literature to find eligible studies focusing on CNS microstructural alterations assessed by DTI and NODDI in COVID-19 patients. Despite some incongruence in the results, there were some important findings, which are as follows. Mean diffusivity and fractional anisotropy were the most reported diffusion parameters. Also, longitudinal fasciculi, thalamic radiations, CC, FOF, CST and UF were repeatedly reported among the significant results in the studies, of which the results on CR and LF were almost consistent. Increased RD in the CC, CR, LF, UF, and FOF (Bispo et al. 2022; Yang et al. 2021; Campabadal et al. 2023; Paolini et al. 2023; Pelizzari et al. 2022; Qin et al. 2024; Arrigoni et al. 2024; Planchuelo-Gómez et al. 2023; Mishra et al. 2024), increased MD in the CC and CR (Silva et al. 2021; Yang et al. 2021; Campabadal et al. 2023; Paolini et al. 2023; Qin et al. 2024; Arrigoni et al. 2024), and decreased AD in the LF and FOF (Bispo et al. 2022; Lu et al. 2020; Díez-Cirarda et al. 2022; Paolini et al. 2023; Pelizzari et al. 2022; Liang et al. 2023; Qin et al. 2024; Planchuelo-Gómez et al. 2023) were the most consistent reported results. The results of NODDI studies were in favor of lower fiber density in left arcuate fasciculus and SLF and lower FD and total AFD in a myriad of tracts including ILF, SLF, CST, and IFOF in Covid-19 patients (Bispo et al. 2022). Network analysis revealed lower local and global efficiencies, and longer shortest path lengths in the superior occipital region of Covid-19 patients (Yang et al. 2021). Also, the results of included studies indicated a pattern of cognitive decline in Covid-19 patients which in some cases, associated with diffusion metrics. For instance, IES-R and BDI scores negatively correlated with AD in the SLF and posterior CR on both sides (Benedetti et al. 2021). Post-traumatic stress disorder scores had positive correlations of FA and negative correlations of RD and MD in several white matter tracts (Yang et al. 2021). Additionally, FA and MD in WM clusters such as CC and cingulum bundle were associated with a decreased cognitive function and TMT results, respectively (Serrano et al. 2024; Petersen et al. 2023).

Viral infections can have impacts on neural microstructure through different mechanisms. Herweh et al. (Herweh et al. 2007) found a decreased FA and an increased MD in patients with Herpes simplex encephalitis due to vasogenic edema. In another study, similar results were observed in the tracts within the medial temporal lobe, indicating an association with memory impairment (Grydeland et al. 2010). Furthermore, Medhi and colleagues (Medhi et al. 2021) found decreased FA values in several WM tracts, such as CC, CR, left posterior thalamic radiation, cingulum, SLF, ILF, IFOF, UF, and fornix. These findings were associated with impaired visual recognition, memory, and auditory verbal learning. Our study highlights a similar pattern of findings, demonstrating alteration in the diffusion parameters in CC and CR, which was found to be correlated with a decline cognitive functions, such as episodic memory, object recognition, and word list recall (Serrano et al. 2024; Petersen et al. 2023).

In the study by Nalbandian et al. (Nalbandian et al. 2021), where they investigated longitudinal changes associated with COVID-19, acute COVID-19 was defined as the initial four weeks following symptom onset during which the PCR test typically yields positive results. Complications that occurred beyond this time frame were referred to as post-acute COVID-19, which is further categorized into two phases: subacute COVID-19, which encompasses symptoms experienced from 4 to 12 weeks after symptom onset, and beyond that timeframe, which is classified as chronic or post- COVID-19 syndrome. Based on the provided information, participants in most of the included studies were in the post-acute COVID-19 phase (Bispo et al. 2022; Benedetti et al. 2021; Huang et al. 2022; Huang et al. 2023; Lu et al. 2020; Qin et al. 2021; Silva et al. 2021; Tian et al. 2022; Yang et al. 2021; Campabadal et al. 2023; Yildirim et al. 2022; Díez-Cirarda et al. 2022; Paolini et al. 2023; Pelizzari et al. 2022; Liang et al. 2023; Chaganti et al. 2024; 2024; Lipton et al. 2024; Petersen et al. 2023; Fineschi et al. 2024; Nelson et al. 2024; Sun et al. 2025; Ibrahim et al. 2024; Teller et al. 2023; Balsak et al. 2023; Scardua-Silva et al. 2024; Boito et al. 2023; Kausel et al. 2024; Trufanov et al. 2025; Mishra et al. 2024). Neuropsychiatric post-COVID-19 condition often associates with attention impairment and processing slowness (Seibert et al. 2025; Akbarzadeh et al. 2025). Post-COVID-19 depression and other symptoms such as anxiety, PTSD, and sleep disturbances are among the frequently reported and have had a tremendous effect on quality of life (Nalbandian et al. 2021; Mazza et al. 2022; Kumar et al. 2025). Consistently, our findings notes higher rates of mental fatigue and depressive symptoms in the COVID-19 patients (Bispo et al. 2022; Silva et al. 2021). Lai et al. (Lai and Wu 2014), found a decreased FA and increased RD in the left and bilateral SLF, respectively, in patients with major depressive disorder (MDD). Similarly, seven studies in our review demonstrated almost the same results (Bispo et al. 2022; Paolini et al. 2023; Pelizzari et al. 2022; 2024; Sun et al. 2025; Deuter et al. 2024; Planchuelo-Gómez et al. 2023). Also, reduced FA in the left posterior thalamic radiation was reported by Hermesdorf et al. (Hermesdorf et al. 2017) in the MDD patients. Consistent with this, Yang and colleagues (Yang et al. 2021) reported similar alterations in their COVID-19 group. On the other hand, O’Doherty et al. (O'Doherty et al. 2018) noted reduced integrity in the CC and UF in patients with PTSD, which was also reported by our included studies. Considering that SLF plays a significant role in connecting different regions in the frontal and parietal lobes, disturbances in some of its functions, such as speech processing and visuospatial orientation, might underlie depressive symptoms (Wang et al. 2022; Janelle et al. 2022). Furthermore, according to the evidence, lower integrity in the left posterior thalamic radiation, CC, and UF in the COVID-19 patients might be the potential underlying cause for their PTSD-like anxiety and depressive symptoms.

Thalamocortical radiations are a group of fiber bundles connecting thalamic nuclei to frontal (anterior) or occipitoparietal (posterior) areas. They are affected by a wide range of neuropsychiatric disorders, such as major depressive disorder, anorexia nervosa, obsessive–compulsive disorder, and negative symptoms of schizophrenia (Lai and Wu 2014; Frieling et al. 2012; Wang et al. 2018). We found that most of the studies were consistent in reporting loss of integrity and disruption in myelination of these bundles in COVID-19 group (Yang et al. 2021; Campabadal et al. 2023; Pelizzari et al. 2022; Sun et al. 2025; Trufanov et al. 2025). Yang and colleagues mentioned significant correlations between DTI findings and PTSD and GAD scores (Yang et al. 2021). These findings suggest that damages in thalamocortical radiations might have taken part in post COVID-19 anxiety and PTSD. Notably, lower FA and higher RD in thalamic radiations are key findings associated with cognitive dysfunction in early stage of Alzheimer disease and mild cognitive impairment, as the integrity of thalamic connections maintain cognitive tasks (Wen et al. 2019; Zhu et al. 2015). Putting all these findings together, we cannot overlook the role that disturbance of thalamocortical radiations plays in cognitive impairments in COVID-19 patients, a finding which might further suggest potential contributions of COVID-19 to neurodegeneration.

Being one of the most prominent WM bundles that connect different interhemispheric regions, CC is involved in several functions. Studies have shown that CC infarction can cause cognitive dysfunction in 40% of cases (Yang et al. 2014). According to the studies by Frederiksen et al. (Frederiksen et al. 2012) and Ryberg et al. (Ryberg et al. 2011), atrophy in the CC, especially in the genu and splenium is correlated with lower Mini-Mental State Examination (MMSE) scores and lower global cognitive functions. Multiple studies have highlighted changes in CC microstructure in post-COVID-19 condition. Pacheco-Jaime et al. (Pacheco-Jaime et al. 2025) found lower FA in the splenium and genu of CC in the post-COVID-19 individuals. Similarly, Ibrahim and colleagues (Klopfenstein et al. 2020) illustrated a significant increase in MD and RD in the body of CC in patients with a history of COVID-19 infection. Despite some inconsistency, most of the studies reported an increase in MD and RD in CC (Silva et al. 2021; Yang et al. 2021; Campabadal et al. 2023; Paolini et al. 2023; Qin et al. 2024; Arrigoni et al. 2024), reflecting disrupted microstructural integrity of this WM tract. Also, Yang et al. (Yang et al. 2021) reported increased MD and RD in the CC and implied an increase in FA and its correlation with PTSD and GAD scores. Accordingly, it can be hypothesized that during the post-COVID phase, disturbances in the CC might underlie cognitive changes. Whether or not such structural changes in CC can contribute to susceptibility to development of neurodegenerative diseases such as Alzheimer disease should be investigated by longitudinal studies. Previous studies focusing on the possible effects of COVID-19 on neurodegeneration have demonstrated that patients suffering from existing neurodegenerative disorders are more susceptible to develop new neuropsychiatric symptoms, such as anxiety and depression (Lingor et al. 2022). Besides, further investigations showed that COVID-19 can induce neurodegeneration through neuroinflammation, microglial activation and leukocyte infiltration. The aforementioned mechanisms induce cytokine production, apoptosis, and disturbances in neurotransmission, leading to a neurodegenerative process. On the other hand, the interaction between the virus and the host proteins might be a potential cause for cellular injury in CNS. Correspondingly, the aforementioned mechanisms such as T cell infiltration have also been detected in the CNS of the patients with neurodegenerative disorders such as Alzheimer disease and Parkinson disease (Dolatshahi et al. 2021). Consequently, we can hypothesize that multiple microstructural alterations in COVID-19 patients can contribute in neurodegeneration and a decline in cognitive function in the future, which has been witnessed by recent studies (Seibert et al. 2025; Akbarzadeh et al. 2025).

Olfactory dysfunction, was one of the early reported neurological symptoms in SARS-CoV-2 patients (Augustin et al. 2021). Intriguingly, a follow-up study found that 12.4% of patients recovering from the acute phase of the disease still had anosmia as a chronic symptom of post-COVID syndrome (Ellul et al. 2020). However, the exact mechanism behind SARS-CoV-2-related anosmia is still scarce. Initially, it was thought that local inflammation and sinusitis were responsible, but subsequent investigations showed that olfactory dysfunction persists even after recovery from other symptoms (Meinhardt et al. 2021). Additionally, some patients with anosmia had no signs of sinusitis in sinus imaging (Meinhardt et al. 2021). Recent evidence suggests that the involvement of olfactory tract and olfactory bulb may play a key role in mediating anosmia in SARS-CoV-2 patients. The virus's genome has been detected in sustentacular cells of the olfactory epithelium, indicating possible axonal transmission (Bilinska et al. 2021). Olfactory epithelial cells express ACE-2 and TMPRSS2, which are crucial for the virus to enter target cells (Karimi-Galougahi et al. 2020). Autopsies of COVID-19 patients revealed viral invasion of the olfactory epithelium and bulb (Bilinska et al. 2021) and PET-CT scans of COVID-19 patients showed reduced activity in the orbitofrontal cortex, a region linked to olfactory sensory processing (Karimi-Galougahi et al. 2020). Yildirim et al. (Yildirim et al. 2022) and Arrigoni et al. (Arrigoni et al. 2024) investigated the DTI findings in the olfactory tract-related regions of Covid-19 patients with persistent olfactory dysfunction. The findings Yildirim and colleagues (Yildirim et al. 2022) revealed a higher quantitative anisotropy value at orbitofrontal, entorhinal region, and orbitofrontal to entorhinal connections in these patients. Also, the authors noticed an asymmetry between connection fibers in the left and right side in 66.7% of COVID-19 patients. Furthermore, Lipton and colleagues demonstrated a decreased ODI in the left orbitofrontal and the right entorhinal gray matters. Entorhinal region provides a cortical connection between olfactory system and hippocampus and the orbitofrontal region acts closely with olfactory tract to process the higher order olfactory inputs. These results might pave the way for future endeavors to shed light on the pathophysiology of Covid-19 related olfactory dysfunction.

Our study had some limitations: First, not all participants in the included studies were in the post-COVID phase. Second, imaging timelines varied vastly between the studies. However, except for two studies which investigated the time overlap between acute and post-acute COVID-19 syndrome, the rest of the studies focused on post-acute COVID-19 and none of the included studies reported data on the acute phase of COVID-19. In order to study the changes in the DTI metrics throughout the disease course (from acute to chronic), longitudinal studies with multiple measurements in different timelines are required. Third, despite all the research, some aspects of COVID-19 are unknown. More studies with long follow-up periods should be performed to delineate the long-term effects of this disease on the CNS.

In conclusion, this systematic review shows that microstructural changes in several white matter tracts in microstructural integrity, especially in WM tracts such as SLF, thalamocortical radiations, and CC, can be correlated with psychiatric and cognitive symptoms in post-COVID-19 phase. Also, novel models such as NODDI and diffusion basis spectrum imaging can be implemented to discover further brain microstructural changes and neuroinflammation in this disease. These findings pave the way for future investigations to provide a better understanding on the pathophysiological basis of the neurological sequelae in COVID-19 patients.

## Supplementary Information

Below is the link to the electronic supplementary material.

Supplementary file1 (DOCX 15 KB)

Supplementary file2 (DOCX 29 KB)

Supplementary file3 (DOCX 25 KB)

## Data Availability

No datasets were generated or analysed during the current study.

## References

[CR1] Akbarzadeh, F., Faridhosseini, F., Eslamzadeh, M., Ghalandarzadeh, M., Hajebikhaniki, S., & Ebrahimi, A. (2025). Cognitive functioning in young adults after mild COVID-19: A case-control study from Iran. *IBRO Neuroscience Reports,**19*, 117–123. 40600171 10.1016/j.ibneur.2025.06.003 PMC12209884

[CR2] Alexander, A. L., Lee, J. E., Lazar, M., & Field, A. S. (2007). Diffusion tensor imaging of the brain. *Neurotherapeutics,**4*(3), 316–329. 17599699 10.1016/j.nurt.2007.05.011 PMC2041910

[CR3] Arab, A., Wojna-Pelczar, A., Khairnar, A., Szabó, N., & Ruda-Kucerova, J. (2018). Principles of diffusion kurtosis imaging and its role in early diagnosis of neurodegenerative disorders. *Brain Research Bulletin,**139*, 91–98. 29378223 10.1016/j.brainresbull.2018.01.015

[CR4] Arrigoni, A., Previtali, M., Bosticardo, S., Pezzetti, G., Poloni, S., Capelli, S., et al. (2024). Brain microstructure and connectivity in COVID-19 patients with olfactory or cognitive impairment. *NeuroImage: Clinical*. 10.1016/j.nicl.2024.103631 38878591 10.1016/j.nicl.2024.103631 PMC11225694

[CR5] Augustin, M., Schommers, P., Stecher, M., Dewald, F., Gieselmann, L., Gruell, H., et al. (2021). Post-COVID syndrome in non-hospitalised patients with COVID-19: A longitudinal prospective cohort study. *The Lancet Regional Health*. 10.1016/j.lanepe.2021.100122 34027514 10.1016/j.lanepe.2021.100122 PMC8129613

[CR6] Balcom, E. F., Nath, A., & Power, C. (2021). Acute and chronic neurological disorders in COVID-19: Potential mechanisms of disease. *Brain,**144*(12), 3576–3588. 34398188 10.1093/brain/awab302 PMC8719840

[CR7] Balsak, S., Atasoy, B., Donmez, Z., Yabul, F. C., Daşkaya, H., Akkoyunlu, Y., et al. (2023). Microstructural alterations in hypoxia-related BRAIN centers after COVID-19 by using DTI: A preliminary study. *Journal of Clinical Ultrasound,**51*(7), 1276–1283. 37293861 10.1002/jcu.23503

[CR8] Basser, P. J., Mattiello, J., & LeBihan, D. (1994). MR diffusion tensor spectroscopy and imaging. *Biophysical Journal,**66*(1), 259–267. 8130344 10.1016/S0006-3495(94)80775-1 PMC1275686

[CR9] Beaulieu, C. (2002). The basis of anisotropic water diffusion in the nervous system - a technical review. *NMR in Biomedicine,**15*(7–8), 435–455. 12489094 10.1002/nbm.782

[CR10] Behrens, T. E. J., Berg, H. J., Jbabdi, S., Rushworth, M. F. S., & Woolrich, M. W. (2007). Probabilistic diffusion tractography with multiple fibre orientations: What can we gain? *NeuroImage,**34*(1), 144–155. 17070705 10.1016/j.neuroimage.2006.09.018 PMC7116582

[CR11] Benedetti, F., Palladini, M., Paolini, M., Melloni, E., Vai, B., De Lorenzo, R., et al. (2021). Brain correlates of depression, post-traumatic distress, and inflammatory biomarkers in COVID-19 survivors: A multimodal magnetic resonance imaging study. *Brain, Behavior, & Immunity - Health,**18*, Article 100387. 10.1016/j.bbih.2021.100387 PMC8562046 34746876

[CR12] Bilinska K, von Bartheld CS, Butowt R. Expression of the ACE2 virus entry protein in the nervus terminalis suggests an alternative route for brain infection in COVID-19. bioRxiv : the preprint server for biology. 2021. 10.3389/fncel.2021.674123 PMC8287262 34290590

[CR13] Bispo, D., Hosp, J., Griianti, L., de Carvalho, Diego, Bispo, D., de Paula, Renato, Brandão, P., Assis Pereira, D., et al. (2022). Brain microstructural changes and fatigue after COVID-19. *Frontier in Neurology.,**13*, 1029302. 10.3389/fneur.2022.1029302 PMC9685991 36438956

[CR14] Boito, D., Eklund, A., Tisell, A., Levi, R., Özarslan, E., & Blystad, I. (2023). MRI with generalized diffusion encoding reveals damaged white matter in patients previously hospitalized for COVID-19 and with persisting symptoms at follow-up. *Brain Communications*. 10.1093/braincomms/fcad284 37953843 10.1093/braincomms/fcad284 PMC10638510

[CR15] Campabadal, A., Oltra, J., Junqué, C., Guillen, N., Botí, M. Á., Sala-Llonch, R., et al. (2023). Structural brain changes in post-acute COVID-19 patients with persistent olfactory dysfunction. *Annals of Clinical and Translational Neurology.,**10*(2), 195–203. 36525472 10.1002/acn3.51710 PMC9878006

[CR16] Catani, M., Howard, R. J., Pajevic, S., & Jones, D. K. (2002). Virtual in vivo interactive dissection of white matter fasciculi in the human brain. *NeuroImage,**17*(1), 77–94. 12482069 10.1006/nimg.2002.1136

[CR17] Chaganti, J., Poudel, G., Cysique, L. A., Dore, G. J., Kelleher, A., Matthews, G., et al. (2024). Blood brain barrier disruption and glutamatergic excitotoxicity in post-acute sequelae of SARS COV-2 infection cognitive impairment: Potential biomarkers and a window into pathogenesis. *Frontiers in Neurology*. 10.3389/fneur.2024.1350848 38756214 10.3389/fneur.2024.1350848 PMC11097901

[CR18] Churchill, N. W., Roudaia, E., Chen, J. J., Sekuler, A., Gao, F., Masellis, M., et al. (2024). Effects of post-acute COVID-19 syndrome on cerebral white matter and emotional health among non-hospitalized individuals. *Frontiers in Neurology*. 10.3389/fneur.2024.1432450 39165270 10.3389/fneur.2024.1432450 PMC11333225

[CR19] Clark, K. A., Nuechterlein, K. H., Asarnow, R. F., Hamilton, L. S., Phillips, O. R., Hageman, N. S., et al. (2011). Mean diffusivity and fractional anisotropy as indicators of disease and genetic liability to schizophrenia. *Journal of Psychiatric Research,**45*(7), 980–988. 21306734 10.1016/j.jpsychires.2011.01.006 PMC3109158

[CR20] Desforges, M., Le Coupanec, A., Dubeau, P., Bourgouin, A., Lajoie, L., Dubé, M., et al. (2019). Human coronaviruses and other respiratory viruses: Underestimated opportunistic pathogens of the central nervous system? *Viruses*. 10.3390/v12010014 31861926 10.3390/v12010014 PMC7020001

[CR21] Deuter, D., Hense, K., Kunkel, K., Vollmayr, J., Schachinger, S., Wendl, C., et al. (2024). SARS-CoV2 evokes structural brain changes resulting in declined executive function. *PLoS ONE*. 10.1371/journal.pone.0298837 38470899 10.1371/journal.pone.0298837 PMC10931481

[CR22] Díez-Cirarda, M., Yus, M., Gómez-Ruiz, N., Polidura, C., Gil-Martínez, L., Delgado-Alonso, C., et al. (2022). Multimodal neuroimaging in post-COVID syndrome and correlation with cognition. *Brain*. 10.1093/brain/awac384 10.1093/brain/awac384 PMC9620345 36288544

[CR23] Dolatshahi, M., Sabahi, M., & Aarabi, M. H. (2021). Pathophysiological clues to how the emergent SARS-CoV-2 can potentially increase the susceptibility to neurodegeneration. *Molecular Neurobiology: Springer*. 10.1007/s12035-020-02236-2 10.1007/s12035-020-02236-2 PMC7791539 33417221

[CR24] Ellul, M. A., Benjamin, L., Singh, B., Lant, S., Michael, B. D., Easton, A., et al. (2020). Neurological associations of COVID-19. *The Lancet Neurology*. 10.1016/S1474-4422(20)30221-0 32622375 10.1016/S1474-4422(20)30221-0 PMC7332267

[CR25] Feldman, H. M., Yeatman, J. D., Lee, E. S., Barde, L. H. F., & Gaman-Bean, S. (2010). Diffusion tensor imaging: A review for pediatric researchers and clinicians. *Journal of Developmental and Behavioral Pediatrics,**31*(4), 346–356. 20453582 10.1097/DBP.0b013e3181dcaa8b PMC4245082

[CR26] Fineschi, S., Fahlström, M., Fällmar, D., Haller, S., & Wikström, J. (2024). Comprehensive MRI assessment reveals subtle brain findings in non-hospitalized post-COVID patients with cognitive impairment. *Frontiers in Neuroscience*. 10.3389/fnins.2024.1435218 39319311 10.3389/fnins.2024.1435218 PMC11420131

[CR27] Frederiksen, K. S., Garde, E., Skimminge, A., Barkhof, F., Scheltens, P., Van Straaten, E. C. W., et al. (2012). Corpus callosum tissue loss and development of motor and global cognitive impairment: The LADIS study. *Dementia and Geriatric Cognitive Disorders.,**32*(4), 279–286. 10.1159/000334949 22262017

[CR28] Frieling, H., Fischer, J., Wilhelm, J., Engelhorn, T., Bleich, S., Hillemacher, T., et al. (2012). Microstructural abnormalities of the posterior thalamic radiation and the mediodorsal thalamic nuclei in females with anorexia nervosa - A voxel based diffusion tensor imaging (DTI) study. *Journal of Psychiatric Research,**46*(9), 1237–1242. 22770509 10.1016/j.jpsychires.2012.06.005

[CR29] Grydeland, H., Walhovd, K. B., Westlye, L. T., Due-Tønnessen, P., Ormaasen, V., Sundseth, Ø., & Fjell, A. M. (2010). Amnesia following herpes simplex encephalitis: Diffusion-tensor imaging uncovers reduced integrity of normal-appearing white matter. *Radiology,**257*(3), 774–781. 20935078 10.1148/radiol.10100179

[CR30] Hermesdorf, M., Berger, K., Szentkirályi, A., Schwindt, W., Dannlowski, U., & Wersching, H. (2017). Reduced fractional anisotropy in patients with major depressive disorder and associations with vascular stiffness. *NeuroImage: Clinical,**14*, 151–155. 28180073 10.1016/j.nicl.2017.01.013 PMC5279701

[CR31] Herweh, C., Hartmann, M., Gass, A., Sellner, J., Heiland, S., Nagel, S., et al. (2007). Quantitative diffusion tensor imaging in herpes simplex virus encephalitis. *Journal of Neurovirology,**13*(5), 426–432. 17994427 10.1080/13550280701456498

[CR32] Huang, S., Zhou, X., Zhao, W., Du, Y., Yang, D., Huang, Y., et al. (2023). Dynamic white matter changes in recovered COVID-19 patients: A two-year follow-up study. *Theranostics,**13*(2), 724–735. 36632218 10.7150/thno.79902 PMC9830428

[CR33] Huang, S., Zhou, Z., Yang, D., Zhao, W., Zeng, M., Xie, X., et al. (2022). Persistent white matter changes in recovered COVID-19 patients at the 1-year follow-up. *Brain : A Journal of Neurology,**145*(5), 1830–1838. 34918020 10.1093/brain/awab435 PMC8754808

[CR34] Ibrahim, I., Škoch, A., Dezortová, M., Adla, T., Flusserová, V., Nagy, M., et al. (2024). Evaluation of microstructural brain changes in post-coronavirus disease 2019 (COVID-19) patients with neurological symptoms: A cross-sectional study. *Quantitative Imaging in Medicine and Surgery,**14*(8), 5499–5512. 39144056 10.21037/qims-24-162 PMC11320515

[CR35] Janelle, F., Iorio-Morin, C., D’Amour, S., & Fortin, D. (2022). Superior Longitudinal Fasciculus: A Review of the Anatomical Descriptions With Functional Correlates. *Frontiers in Neurology. *Frontiers Media S.A. 10.3389/fneur.2022.794618 PMC9093186 35572948

[CR36] Karimi-Galougahi, M., Yousefi-Koma, A., Bakhshayeshkaram, M., Raad, N., & Haseli, S. (2020). 18FDG PET/CT scan reveals hypoactive orbitofrontal cortex in anosmia of COVID-19. *Academic Radiology,**27*(7), 1042–1043. 32386948 10.1016/j.acra.2020.04.030 PMC7196385

[CR37] Kausel, L., Figueroa-Vargas, A., Zamorano, F., Stecher, X., Aspé-Sánchez, M., Carvajal-Paredes, P., et al. (2024). Patients recovering from COVID-19 who presented with anosmia during their acute episode have behavioral, functional, and structural brain alterations. *Scientific Reports*. 10.1038/s41598-024-69772-y 39152190 10.1038/s41598-024-69772-y PMC11329703

[CR38] Klopfenstein, T., Kadiane-Oussou, N. J., Toko, L., Royer, P. Y., Lepiller, Q., Gendrin, V., et al. (2020). Features of anosmia in COVID-19. *Médecine Et Maladies Infectieuses,**50*(5), 436–439. 32305563 10.1016/j.medmal.2020.04.006 PMC7162775

[CR39] Kumar, N., Lam, C. N., Lee, R., Unger, J. B., & Sood, N. (2025). The association between baseline physical and mental health and the risk of postacute sequelae of COVID-19 infection. *Scientific Reports,**15*(1), Article 24374. 40628861 10.1038/s41598-025-09676-7 PMC12238242

[CR40] Lai, C. H., & Wu, Y. T. (2014). Alterations in white matter micro-integrity of the superior longitudinal fasciculus and anterior thalamic radiation of young adult patients with depression. *Psychological Medicine,**44*(13), 2825–2832. 25065445 10.1017/S0033291714000440

[CR41] Li, C., Liu, J., Lin, J., & Shang, H. (2022). COVID-19 and risk of neurodegenerative disorders: A Mendelian randomization study. *Translational Psychiatry*. 10.1038/s41398-022-02052-3 35835752 10.1038/s41398-022-02052-3 PMC9281279

[CR42] Liang, H., Ernst, T., Oishi, K., Ryan, M. C., Herskovits, E., Cunningham, E., et al. (2023). Abnormal brain diffusivity in participants with persistent neuropsychiatric symptoms after COVID-19. *NeuroImmune Pharmacology and Therapeutics.,**2*, 37. 37067870 10.1515/nipt-2022-0016 PMC10091517

[CR43] Lingor, P., Demleitner, A. F., Wolff, A. W., & Feneberg, E. (2022). SARS-CoV-2 and neurodegenerative diseases: What we know and what we don’t. *Journal of Neural Transmission*. 10.1007/s00702-022-02500-w 35434769 10.1007/s00702-022-02500-w PMC9013492

[CR44] Lipton, M. L., Fleysher, R., Song, J. Y., Ye, K., Zimmerman, M. E., Lipton, R. B., et al. (2024). Brain effects of mild COVID-19 in healthy young adults: A pilot study. *Heliyon*. 10.1016/j.heliyon.2024.e34764 39157305 10.1016/j.heliyon.2024.e34764 PMC11327499

[CR45] Lu, Y., Li, X., Geng, D., Mei, N., Wu, P.-Y., Huang, C.-C., et al. (2020). Cerebral micro-structural changes in COVID-19 patients - An MRI-based 3-month follow-up study. *EClinicalMedicine,**25*, Article 100484. 32838240 10.1016/j.eclinm.2020.100484 PMC7396952

[CR46] Mao, L., Jin, H., Wang, M., Hu, Y., Chen, S., He, Q., et al. (2020). Neurologic manifestations of hospitalized patients with Coronavirus disease 2019 in Wuhan, China. *JAMA Neurology,**77*(6), 683–690. 32275288 10.1001/jamaneurol.2020.1127 PMC7149362

[CR47] Mazza, M. G., Palladini, M., Poletti, S., & Benedetti, F. (2022). Post-COVID-19 depressive symptoms: Epidemiology, pathophysiology, and pharmacological treatment. *CNS Drugs* (pp. 681–702). Adis. 10.1007/s40263-022-00931-3 PMC9210800 35727534

[CR48] Medhi, G., Kapadia, A., Parida, S., Dhanya, C., Bagepalli, B. S., Netravathi, M., et al. (2021). Long-term sequelae of herpes simplex virus encephalitis–related white matter injury: Correlation of neuropsychological outcome and diffusion tensor imaging. *Journal of NeuroVirology.,**27*(4), 601–608. 34398444 10.1007/s13365-021-01000-z

[CR49] Meinhardt, J., Radke, J., Dittmayer, C., Franz, J., Thomas, C., Mothes, R., et al. (2021). Olfactory transmucosal SARS-CoV-2 invasion as a port of central nervous system entry in individuals with COVID-19. *Nature Neuroscience.,**24*(2), 168–175. 33257876 10.1038/s41593-020-00758-5

[CR50] Mishra, S. S., Pedersini, C. A., Misra, R., Gandhi, T. K., Rokers, B., & Biswal, B. B. (2024). Tracts in the limbic system show microstructural alterations post COVID-19 recovery. *Brain Communications*. 10.1093/braincomms/fcae139 38715715 10.1093/braincomms/fcae139 PMC11074789

[CR51] Nalbandian, A., Sehgal, K., Gupta, A., Madhavan, M. V., McGroder, C., Stevens, J. S., et al. (2021). Post-acute COVID-19 syndrome. *Nature Medicine*. 10.1038/s41591-021-01283-z 33753937 10.1038/s41591-021-01283-z PMC8893149

[CR52] Nelson, B. K., Farah, L. N., Grier, A., Su, W., Chen, J., Sossi, V., et al. (2024). Differences in brain structure and cognitive performance between patients with long-COVID and those with normal recovery. *NeuroImage*. 10.1016/j.neuroimage.2024.120859 39317274 10.1016/j.neuroimage.2024.120859

[CR53] Nygaard, M. K. E., Riemenschneider, M., Gaemelke, T., Dalgas, U., & Eskildsen, S. F. (2025). Spatiotemporal alterations of gray matter microstructure in newly diagnosed relapsing-remitting multiple sclerosis patients: A longitudinal diffusion kurtosis MRI study. *Journal of the Neurological Sciences*. 10.1016/j.jns.2025.123551 40411942 10.1016/j.jns.2025.123551

[CR54] O’Doherty, D. C. M., Ryder, W., Paquola, C., Tickell, A., Chan, C., Hermens, D. F., et al. (2018). White matter integrity alterations in post-traumatic stress disorder. *Human Brain Mapping.,**39*(3), 1327–1338. 29265681 10.1002/hbm.23920 PMC6866495

[CR55] Pacheco-Jaime, L., Garcia-Vicente, C., Ariza, M., Cano, N., Garolera, M., Carreras-Vidal, L., et al. (2025). Structural brain changes in post-COVID condition and its relationship with cognitive impairment. *Brain Communications,**7*(1), Article fcaf070. 40008326 10.1093/braincomms/fcaf070 PMC11851114

[CR56] Page, M. J., McKenzie, J. E., Bossuyt, P. M., Boutron, I., Hoffmann, T. C., Mulrow, C. D., et al. (2021). *The PRISMA 2020 statement: An updated guideline for reporting systematic reviews*. BMJ Publishing Group. 10.1136/bmj.n71 PMC8005924 33782057

[CR57] Paolini, M., Palladini, M., Mazza, M. G., Colombo, F., Vai, B., Rovere-Querini, P., et al. (2023). Brain correlates of subjective cognitive complaints in COVID-19 survivors: A multimodal magnetic resonance imaging study. *European Neuropsychopharmacology,**68*, 1–10. 36640728 10.1016/j.euroneuro.2022.12.002 PMC9742225

[CR58] Pelizzari, L., Cazzoli, M., Lipari, S., Laganà, M. M., Cabinio, M., Isernia, S., et al. (2022). Mid-term MRI evaluation reveals microstructural white matter alterations in COVID-19 fully recovered subjects with anosmia presentation. *Therapeutic Advances in Neurological Disorders,**15*, Article 17562864221111996. 10.1177/17562864221111995 PMC9310254 35899101

[CR59] Petersen, M., Nägele, F. L., Mayer, C., Schell, M., Petersen, E., Kühn, S., et al. (2023). Brain imaging and neuropsychological assessment of individuals recovered from a mild to moderate SARS-CoV-2 infection. *Proceedings of the National Academy of Sciences of the United States of America*. 10.1073/pnas.2217232120 37220275 10.1073/pnas.2217232120 PMC10235949

[CR60] Pierpaoli, C., & Basser, P. J. (1996). Toward a quantitative assessment of diffusion anisotropy. *Magnetic Resonance in Medicine,**36*(6), 893–906. 8946355 10.1002/mrm.1910360612

[CR61] Planchuelo-Gómez, Á., García-Azorín, D., Guerrero, Á. L., Rodríguez, M., Aja-Fernández, S., & de Luis-García, R. (2023). Structural brain changes in patients with persistent headache after COVID-19 resolution. *Journal of Neurology,**270*(1), 13–31. 36178541 10.1007/s00415-022-11398-z PMC9522538

[CR62] Preziosa, P., Pagani, E., Meani, A., Marchesi, O., Conti, L., Falini, A., et al. (2023). NODDI, diffusion tensor microstructural abnormalities and atrophy of brain white matter and gray matter contribute to cognitive impairment in multiple sclerosis. *Journal of Neurology.,**270*(2), 810–823. 36201016 10.1007/s00415-022-11415-1

[CR63] Qin, H., Duan, G., Zhou, K., Qin, L., Lai, Y., Liu, Y., et al. (2024). Alteration of white matter microstructure in patients with sleep disorders after COVID-19 infection. *Sleep Medicine,**114*, 109–118. 38181582 10.1016/j.sleep.2023.12.024

[CR64] Qin, Y., Wu, J., Chen, T., Li, J., Zhang, G., Wu, D., et al. (2021). Long-term microstructure and cerebral blood flow changes in patients recovered from COVID-19 without neurological manifestations. *The Journal of clinical investigation.,**131*, 8. 10.1172/JCI147329 PMC8262559 33630760

[CR65] Romoli, M., Jelcic, I., Bernard-Valnet, R., García Azorín, D., Mancinelli, L., Akhvlediani, T., et al. (2020). A systematic review of neurological manifestations of SARS-CoV-2 infection: The devil is hidden in the details. *European Journal of Neurology,**27*(9), 1712–1726. 32503088 10.1111/ene.14382 PMC7300895

[CR66] Ryberg, C., Rostrup, E., Paulson, O. B., Barkhof, F., Scheltens, P., Van Straaten, E. C. W., et al. (2011). Corpus callosum atrophy as a predictor of age-related cognitive and motor impairment: A 3-year follow-up of the LADIS study cohort. *Journal of the Neurological Sciences,**307*(1–2), 100–105. 21621224 10.1016/j.jns.2011.05.002

[CR67] Sacco, S., Caverzasi, E., Papinutto, N., Cordano, C., Bischof, A., Gundel, T., et al. (2020). Neurite orientation dispersion and density imaging for assessing acute inflammation and lesion evolution in MS. *American Journal of Neuroradiology,**41*(12), 2219–2226. 33154077 10.3174/ajnr.A6862 PMC7963254

[CR68] Sanjari Moghaddam, H., Ghazi Sherbaf, F., & Aarabi, M. H. (2019). Brain microstructural abnormalities in type 2 diabetes mellitus: A systematic review of diffusion tensor imaging studies. *Frontiers in Neuroendocrinology,**55*, Article 100782. 31401292 10.1016/j.yfrne.2019.100782

[CR69] Scardua-Silva, L., da Amorim Costa, B., Karmann Aventurato, Í., Batista Joao, R., de Machado Campos, B., de Rabelo Brito, M., et al. (2024). Microstructural brain abnormalities, fatigue, and cognitive dysfunction after mild COVID-19. *Scientific Reports*. 10.1038/s41598-024-52005-7 38242927 10.1038/s41598-024-52005-7 PMC10798999

[CR70] Seibert, S., Eckert, I., Widmann, C. N., Ebrahimi, T., Bösl, F., Franke, C., et al. (2025). Validity of the test for attentional performance in neurological post-COVID condition. *Science and Reports,**15*(1), Article 24208. 10.1038/s41598-025-09128-2 PMC12234741 40624269

[CR71] Serrano del Pueblo, V. M., Serrano-Heras, G., Romero Sánchez, C. M., Piqueras Landete, P., Rojas-Bartolome, L., Feria, I., et al. (2024). Brain and cognitive changes in patients with long COVID compared with infection-recovered control subjects. *Brain.,**147*, 3611. 38562097 10.1093/brain/awae101

[CR72] Silva LS, Joao RB, Nogueira MH, Aventurato IK, De Campos BM, De Brito MR, et al. Functional and microstructural brain abnormalities, fatigue, and cognitive dysfunction after mild COVID-19. Cold Spring Harbor Laboratory; 2021.

[CR73] Smith, S. M., Jenkinson, M., Johansen-Berg, H., Rueckert, D., Nichols, T. E., Mackay, C. E., et al. (2006). Tract-based spatial statistics: Voxelwise analysis of multi-subject diffusion data. *NeuroImage,**31*(4), 1487–1505. 16624579 10.1016/j.neuroimage.2006.02.024

[CR74] Sporns, O. (2011). The human connectome: A complex network. *Annals of the New York Academy of Sciences*. 10.1111/j.1749-6632.2010.05888.x 21251014 10.1111/j.1749-6632.2010.05888.x

[CR75] Sun, T., Jiang, C., Zhang, Y., Li, Y., Chen, G., Zhou, Y., et al. (2025). Distinguished multimodal imaging features affected by COVID-19 in major depressive disorder patients. *Journal of Psychiatric Research,**183*, 1–9. 39908714 10.1016/j.jpsychires.2025.01.053

[CR76] Teller, N., Chad, J. A., Wong, A., Gunraj, H., Ji, X., Goubran, M., et al. (2023). Feasibility of diffusion-tensor and correlated diffusion imaging for studying white-matter microstructural abnormalities: Application in COVID-19. *Human Brain Mapping,**44*(10), 3998–4010. 37162380 10.1002/hbm.26322 PMC10258529

[CR77] Tian, T., Wu, J., Chen, T., Li, J., Yan, S., Zhou, Y., et al. (2022). Long-term follow-up of dynamic brain changes in patients recovered from COVID-19 without neurological manifestations. *JCI insight.,**7*, 4. 10.1172/jci.insight.155827 PMC8876627 35191397

[CR78] Trufanov A, Voznyuk I, Kutkova A, Efimtsev A, Shusharina N, Ovdienko O. Structural and functional changes in the brain during post-COVID syndrome: neuropsychological and MRI study. European Physical Journal: Special Topics. 2025.

[CR79] van Lith, T. J., Li, H., van der Wijk, M. W., Wijers, N. T., Sluis, W. M., Wermer, M. J. H., et al. (2024). White matter integrity in hospitalized COVID-19 patients is not associated with short- and long-term clinical outcomes. *Frontiers in Neurology*. 10.3389/fneur.2024.1440294 39175757 10.3389/fneur.2024.1440294 PMC11340528

[CR80] Viswanathan M, Ansari MT, Berkman ND, Chang S, Hartling L, McPheeters M, et al. Assessing the Risk of Bias of Individual Studies in Systematic Reviews of Health Care Interventions. 2008. 22479713

[CR81] Wakana, S., Jiang, H., Nagae-Poetscher, L. M., Van Zijl, P. C. M., & Mori, S. (2004). Fiber tract-based atlas of human white matter anatomy. *Radiology,**230*(1), 77–87. 14645885 10.1148/radiol.2301021640

[CR82] Wang, M., Qi, X., Yang, X., Fan, H., Dou, Y., Guo, W., et al. (2022). The pattern glare and visual memory are disrupted in patients with major depressive disorder. *BMC Psychiatry,**22*(1), Article 518-. 35918667 10.1186/s12888-022-04167-9 PMC9344705

[CR83] Wang, R., Fan, Q., Zhang, Z., Chen, Y., Zhu, Y., & Li, Y. (2018). Anterior thalamic radiation structural and metabolic changes in obsessive-compulsive disorder: A combined DTI-MRS study. *Psychiatry Research - Neuroimaging,**277*, 39–44. 29807209 10.1016/j.pscychresns.2018.05.004

[CR84] Wen, Q., Mustafi, S. M., Li, J., Risacher, S. L., Tallman, E., Brown, S. A., et al. (2019). White matter alterations in early-stage Alzheimer’s disease: A tract-specific study. *Alzheimer’s & Dementia: Diagnosis, Assessment & Disease Monitoring,**11*, 576–587. 10.1016/j.dadm.2019.06.003 PMC6713788 31467968

[CR85] WHO Coronavirus (COVID-19) Dashboard | WHO Coronavirus (COVID-19) Dashboard With Vaccination Data.

[CR86] Yang, L.-L., Huang, Y.-N., & Cui, Z.-T. (2014). Clinical features of acute corpus callosum infarction patients. *International Journal of Clinical and Experimental Pathology,**7*, 5160. 25197390 PMC4152080

[CR87] Yang, L., Zhou, M., Li, L., Luo, P., Fan, W., Xu, J., et al. (2021). Characteristics of mental health implications and plasma metabolomics in patients recently recovered from COVID-19. *Translational Psychiatry,**11*(1), Article 307. 34021116 10.1038/s41398-021-01426-3 PMC8138845

[CR88] Yildirim, D., Kandemirli, S. G., Tekcan Sanli, D. E., Akinci, O., & Altundag, A. (2022). A comparative olfactory MRI, DTI and fMRI study of COVID-19 related anosmia and post viral olfactory dysfunction. *Academic Radiology,**29*(1), 31–41. 34810059 10.1016/j.acra.2021.10.019 PMC8549400

[CR89] Zhang, H., Schneider, T., Wheeler-Kingshott, C. A., & Alexander, D. C. (2012). NODDI: Practical in vivo neurite orientation dispersion and density imaging of the human brain. *NeuroImage,**61*(4), 1000–1016. 22484410 10.1016/j.neuroimage.2012.03.072

[CR90] Zhu, Q.-Y., Bi, S.-W., Yao, X.-T., Ni, Z.-Y., Li, Y., Chen, B.-Y., et al. (2015). Disruption of thalamic connectivity in Alzheimer’s disease: A diffusion tensor imaging study. *Metabolic Brain Disease,**30*(5), 1295–1308. 26141074 10.1007/s11011-015-9708-7

