# Supplementary material for: Brain microstructural alterations in COVID-19: a systematic review of diffusion weighted imaging studies
Source: Brain Imaging Behav. 2026 Mar 14;20(2):49. doi: 10.1007/s11682-026-01084-3 (PMC12988982; doi:10.1007/s11682-026-01084-3)
Supplement: Supplementary file 1 — Supplementary file1 (DOCX 15 KB) [file 11682_2026_1084_MOESM1_ESM.docx]

Supplementary Table S1. Search strategies database

| **Database** | **Search string** | **Number of results** |
| --- | --- | --- |
| **PubMed** | "Diffusion Tensor Imaging" OR "diffusion weighted magnetic resonance imaging" OR "Diffusion Tractography" OR "diffusion weighted imaging" OR "diffusion weighted MRI" OR "DTI" OR "white matter" OR "grey matter" OR "gray matter" OR "NODDI" OR “Neurite Density" OR “Orientation Dispersion" OR "neurite orientation dispersion and density imaging" OR "diffusion kurtosis imaging" OR "diffusional kurtosis" OR "diffusion kurtosi*" OR "DKI") AND ("COVID 19" OR "COVID-19 Virus Disease" OR "COVID 19 Virus Disease" OR "COVID-19 Virus Diseases" OR "Disease, COVID-19 Virus" OR "Virus Disease, COVID-19" OR "COVID-19 Virus Infection" OR "COVID 19 Virus Infection" OR "COVID-19 Virus Infections" OR "Infection, COVID-19 Virus" OR "Virus Infection, COVID-19" OR "2019-nCoV Infection" OR "2019 nCoV Infection" OR "2019-nCoV Infections" OR "Infection, 2019-nCoV" OR "Coronavirus Disease-19" OR "Coronavirus Disease 19" OR "2019 Novel Coronavirus Disease" OR "2019 Novel Coronavirus Infection" OR "2019-nCoV Disease" OR "2019 nCoV Disease" OR "2019-nCoV Diseases" OR "Disease, 2019-nCoV" OR "COVID19" OR "Coronavirus Disease 2019" OR "Disease 2019, Coronavirus" OR "SARS Coronavirus 2 Infection" OR "SARS-CoV-2 Infection" OR "Infection, SARS-CoV-2" OR "SARS CoV 2 Infection" OR "SARS-CoV-2 Infections" OR "COVID-19 Pandemic" OR "COVID 19 Pandemic" OR "COVID-19 Pandemics" OR "Pandemic, COVID-19" | 591 |
| **Scopus** | "Diffusion Tensor Imaging" OR "diffusion weighted magnetic resonance imaging" OR "Diffusion Tractography" OR "diffusion weighted imaging" OR "diffusion weighted MRI" OR "DTI" OR "white matter" OR "grey matter" OR "gray matter" OR "NODDI" OR “Neurite Density" OR “Orientation Dispersion" OR "neurite orientation dispersion and density imaging" OR "diffusion kurtosis imaging" OR "diffusional kurtosis" OR "diffusion kurtosi*" OR "DKI") AND ("COVID 19" OR "COVID-19 Virus Disease" OR "COVID 19 Virus Disease" OR "COVID-19 Virus Diseases" OR "Disease, COVID-19 Virus" OR "Virus Disease, COVID-19" OR "COVID-19 Virus Infection" OR "COVID 19 Virus Infection" OR "COVID-19 Virus Infections" OR "Infection, COVID-19 Virus" OR "Virus Infection, COVID-19" OR "2019-nCoV Infection" OR "2019 nCoV Infection" OR "2019-nCoV Infections" OR "Infection, 2019-nCoV" OR "Coronavirus Disease-19" OR "Coronavirus Disease 19" OR "2019 Novel Coronavirus Disease" OR "2019 Novel Coronavirus Infection" OR "2019-nCoV Disease" OR "2019 nCoV Disease" OR "2019-nCoV Diseases" OR "Disease, 2019-nCoV" OR "COVID19" OR "Coronavirus Disease 2019" OR "Disease 2019, Coronavirus" OR "SARS Coronavirus 2 Infection" OR "SARS-CoV-2 Infection" OR "Infection, SARS-CoV-2" OR "SARS CoV 2 Infection" OR "SARS-CoV-2 Infections" OR "COVID-19 Pandemic" OR "COVID 19 Pandemic" OR "COVID-19 Pandemics" OR "Pandemic, COVID-19" | 1380 |
| **Web of Science** | "Diffusion Tensor Imaging" OR "diffusion weighted magnetic resonance imaging" OR "Diffusion Tractography" OR "diffusion weighted imaging" OR "diffusion weighted MRI" OR "DTI" OR "white matter" OR "grey matter" OR "gray matter" OR "NODDI" OR “Neurite Density" OR “Orientation Dispersion" OR "neurite orientation dispersion and density imaging" OR "diffusion kurtosis imaging" OR "diffusional kurtosis" OR "diffusion kurtosi*" OR "DKI") AND ("COVID 19" OR "COVID-19 Virus Disease" OR "COVID 19 Virus Disease" OR "COVID-19 Virus Diseases" OR "Disease, COVID-19 Virus" OR "Virus Disease, COVID-19" OR "COVID-19 Virus Infection" OR "COVID 19 Virus Infection" OR "COVID-19 Virus Infections" OR "Infection, COVID-19 Virus" OR "Virus Infection, COVID-19" OR "2019-nCoV Infection" OR "2019 nCoV Infection" OR "2019-nCoV Infections" OR "Infection, 2019-nCoV" OR "Coronavirus Disease-19" OR "Coronavirus Disease 19" OR "2019 Novel Coronavirus Disease" OR "2019 Novel Coronavirus Infection" OR "2019-nCoV Disease" OR "2019 nCoV Disease" OR "2019-nCoV Diseases" OR "Disease, 2019-nCoV" OR "COVID19" OR "Coronavirus Disease 2019" OR "Disease 2019, Coronavirus" OR "SARS Coronavirus 2 Infection" OR "SARS-CoV-2 Infection" OR "Infection, SARS-CoV-2" OR "SARS CoV 2 Infection" OR "SARS-CoV-2 Infections" OR "COVID-19 Pandemic" OR "COVID 19 Pandemic" OR "COVID-19 Pandemics" OR "Pandemic, COVID-19" | 211 |
| **EMBASE** | "Diffusion Tensor Imaging" OR "diffusion weighted magnetic resonance imaging" OR "Diffusion Tractography" OR "diffusion weighted imaging" OR "diffusion weighted MRI" OR "DTI" OR "white matter" OR "grey matter" OR "gray matter" OR "NODDI" OR “Neurite Density" OR “Orientation Dispersion" OR "neurite orientation dispersion and density imaging" OR "diffusion kurtosis imaging" OR "diffusional kurtosis" OR "diffusion kurtosi*" OR "DKI") AND ("COVID 19" OR "COVID-19 Virus Disease" OR "COVID 19 Virus Disease" OR "COVID-19 Virus Diseases" OR "Disease, COVID-19 Virus" OR "Virus Disease, COVID-19" OR "COVID-19 Virus Infection" OR "COVID 19 Virus Infection" OR "COVID-19 Virus Infections" OR "Infection, COVID-19 Virus" OR "Virus Infection, COVID-19" OR "2019-nCoV Infection" OR "2019 nCoV Infection" OR "2019-nCoV Infections" OR "Infection, 2019-nCoV" OR "Coronavirus Disease-19" OR "Coronavirus Disease 19" OR "2019 Novel Coronavirus Disease" OR "2019 Novel Coronavirus Infection" OR "2019-nCoV Disease" OR "2019 nCoV Disease" OR "2019-nCoV Diseases" OR "Disease, 2019-nCoV" OR "COVID19" OR "Coronavirus Disease 2019" OR "Disease 2019, Coronavirus" OR "SARS Coronavirus 2 Infection" OR "SARS-CoV-2 Infection" OR "Infection, SARS-CoV-2" OR "SARS CoV 2 Infection" OR "SARS-CoV-2 Infections" OR "COVID-19 Pandemic" OR "COVID 19 Pandemic" OR "COVID-19 Pandemics" OR "Pandemic, COVID-19" | 627 |
