# Supplementary material for: Brain microstructural alterations in COVID-19: a systematic review of diffusion weighted imaging studies
Source: Brain Imaging Behav. 2026 Mar 14;20(2):49. doi: 10.1007/s11682-026-01084-3 (PMC12988982; doi:10.1007/s11682-026-01084-3)
Supplement: Supplementary file 2 — Supplementary file2 (DOCX 29 KB) [file 11682_2026_1084_MOESM2_ESM.docx]

Supplementary Table S2.1. Quality assessment (Case-control studies)

|  | **Selection** | | | | | **Comparability** | | | | **Exposure** | | |
| --- | --- | --- | --- | --- | --- | --- | --- | --- | --- | --- | --- | --- |
| Study first author/year | Cause definition adequacy | Representative of cases | Selection of controls | Definition of controls (healthy) | Subtotal | Age | Education | Sex | Subtotal | Ascertainment of exposure | Non-response rate | Subtotal |
| Campabadal/2022 | * | * | * | * | 4 | - | - | - | 0 | * | * | 2 |
| Bispo/2022 | * | * | * | * | 4 | * | - | * | 2 | * | * | 2 |
| Huang/2023 | * | * | * | * | 4 | - | - | * | 1 | * | * | 2 |
| Diez-Cirarda/2022 | * | * | * | * | 4 | * | - | - | 1 | * | * | 2 |
| Pelazzari/2022 | * | * | * | * | 4 | * | - | * | 2 | * | * | 2 |
| Tian/2022 | * | * | * | * | 4 | * | * | * | 3 | * | * | 2 |
| Qin/2021 | * | * | * | * | 4 | * | * | * | 3 | * | * | 2 |
| Huang/2021 | * | * | * | * | 4 | * | * | * | 3 | * | * | 2 |
| Yang/2021 | * | * | * | * | 4 | * | * | * | 3 | * | * | 2 |
| Yildrim/2021 | * | * | * | * | 4 | - | - | - | 0 | * | * | 2 |
| Silva/2020 | * | * | * | * | 4 | * | - | * | 2 | * | * | 2 |
| Liang/2023 | * | * | * | * | 4 | * | * | * | 3 | * | * | 2 |
| Qin/2024 | * | * | * | * | 4 | * | * | * | 3 | * | * | 2 |
| Serrano del pueblo/2024 | * | * | * | * | 4 | * | * | * | 3 | * | * | 2 |
| Lipton/2024 | * | * | * | * | 4 | - | - | - | 0 | * | * | 2 |
| Petersen/2023 | * | * | * | * | 4 | * | * | * | 3 | * | * | 2 |
| Arrigoni/2024 | * | * | * | * | 4 | * | - | * | 2 | * | * | 2 |
| Fineschi/2024 | * | * | * | * | 4 | * | - | - | 1 | * | * | 2 |
| Nelson/2024 | * | * | * | * | 4 | * | - | * | 2 | * | * | 2 |
| Sun/2025 | * | * | * | * | 4 | * | - | * | 2 | * | * | 2 |
| W.Churchill/2024 | * | * | * | - | 3 | - | - | - | 0 | * | * | 2 |
| Ibrahim/2024 | * | * | * | * | 4 | * | - | - | 1 | * | * | 2 |
| Teller/2023 | * | * | * | * | 4 | * | - | * | 2 | * | * | 2 |
| Balsak/2023 | * | * | * | * | 4 | - | - | - | 0 | * | * | 2 |
| Scardua‐Silva/2024 | * | * | * | * | 4 | * | - | * | 2 | * | * | 2 |
| Boito et al/2023 | * | * | * | * | 4 | * | - | - | 1 | * | * | 2 |
| Trufanov et al/2024 | * | * | * | * | 4 | - | - | - | 0 | * | * | 2 |
| Planchuelo-Gomez/2023 | * | * | * | * | 4 | * | - | * | 2 | * | * | 2 |
| Mishra et al/2024 | * | * | * | * | 4 | - | - | - | 0 | * | * | 2 |
| Lith/2024 | * | * | * | * | 4 | * | - | * | 2 | * | * | 2 |

|  | | **Selection** | | | | | **Comparability** | | | | **Exposure** | | | |
| --- | --- | --- | --- | --- | --- | --- | --- | --- | --- | --- | --- | --- | --- | --- |
| Study first author/year | Cause definition adequacy | | Representative of cases | Selection of controls | Definition of controls (healthy) | Subtotal | Age | Education | Sex | Subtotal | Assessment of outcome | Follow-up long enough | Adequacy of follow up | Subtotal |
| Paolini/2022 | * | | * | * | * | 4 | * | - | * | 2 | * | * | * | 3 |
| Rau/2022 | * | | * | * | * | 4 | * | - | * | 2 | * | * | * | 3 |
| Benedetti/2021 | * | | * | - | - | 3 | * | - | * | 2 | * | * | * | 3 |
| Lu/2020 | * | | * | * | * | 4 | * | - | * | 2 | * | * | * | 3 |
| Chaganti/2024 | * | | * | * | * | 4 | * | - | * | 2 | * | * | * | 3 |
| Kausel/2024 | * | | * | * | * | 4 | - | - | - | 0 | * | * | * | 3 |
| Deuter/2024 | * | | * | * | * | 4 | * | - | * | 2 | * | * | * | 3 |

Supplementary Table S2.2. Quality assessment (Cohort studies)
