# Supplementary material for: Brain microstructural alterations in COVID-19: a systematic review of diffusion weighted imaging studies
Source: Brain Imaging Behav. 2026 Mar 14;20(2):49. doi: 10.1007/s11682-026-01084-3 (PMC12988982; doi:10.1007/s11682-026-01084-3)
Supplement: Supplementary file 3 — Supplementary file3 (DOCX 25 KB) [file 11682_2026_1084_MOESM3_ESM.docx]

Supplementary Table S3. Risk of bias in included studies.

| Study | **Selection bias** | | **Performance bias** | **Attrition bias** | **Detection bias** | | | | **Reporting bias** |
| --- | --- | --- | --- | --- | --- | --- | --- | --- | --- |
|  | **Q1** | **Q2** | **Q3** | **Q4** | **Q5** | **Q6** | **Q7** | **Q8** | **Q9** |
| Campabadal 2022 | Yes | Yes | Yes | Yes | No | Yes | Yes | Yes | Yes |
| Bispo 2022 | Yes | Yes | Yes | Yes | No | Yes | Yes | Yes | Yes |
| Huang 2023 | Yes | Yes | Yes | Yes | Yes | Yes | Yes | Yes | Yes |
| Diez-Cirarda 2022 | Yes | Yes | Yes | Yes | Yes | Yes | Yes | Yes | Yes |
| Paolini 2022 | Yes | Yes | Yes | Yes | No | Yes | Yes | Yes | Yes |
| Pelazzari 2022 | Yes | Yes | Yes | Yes | No | Yes | Yes | Yes | Yes |
| Rau 2022 | Yes | Yes | Yes | Yes | No | Yes | Yes | Yes | Yes |
| Tian 2022 | Yes | Yes | No | Yes | No | Yes | Yes | Yes | Yes |
| Qin 2021 | Yes | Yes | Yes | Yes | No | Yes | Yes | Yes | Yes |
| Huang 2021 | Yes | Yes | Yes | Yes | No | Yes | Yes | Yes | Yes |
| Benedetti 2021 | Yes | No | Yes | Yes | No | Yes | Yes | No | Yes |
| Yang 2021 | Yes | Yes | Yes | Yes | No | Yes | Yes | Yes | Yes |
| Yildrim 2021 | Yes | No | Yes | Yes | Yes | Yes | Yes | No | Yes |
| Silva 2020 | Yes | Yes | Yes | Yes | No | Yes | Yes | Yes | Yes |
| Lu 2020 | Yes | Yes | No | Yes | No | Yes | Yes | Yes | Yes |
| Liang 2023 | Yes | Yes | Yes | Yes | No | Yes | Yes | Yes | Yes |
| Qin/2024 | Yes | Yes | Yes | Yes | No | Yes | Yes | Yes | Yes |
| Chaganti/2024 | Yes | Yes | Yes | Yes | No | Yes | Yes | Yes | Yes |
| Serrano del pueblo/2024 | Yes | Yes | Yes | Yes | No | Yes | Yes | Yes | Yes |
| Lipton/2024 | Yes | Yes | Yes | Yes | No | Yes | Yes | Yes | Yes |
| Petersen/2023 | Yes | Yes | Yes | Yes | No | Yes | Yes | Yes | Yes |
| Arrigoni/2024 | Yes | Yes | Yes | Yes | No | Yes | Yes | Yes | Yes |
| Fineschi/2024 | Yes | Yes | Yes | Yes | No | Yes | Yes | Yes | Yes |
| Nelson/2024 | Yes | Yes | Yes | Yes | No | Yes | Yes | Yes | Yes |
| Sun/2025 | Yes | Yes | Yes | Yes | No | Yes | Yes | Yes | Yes |
| W.Churchill/2024 | Yes | Yes | Yes | Yes | No | Yes | Yes | Yes | Yes |
| Ibrahim/2024 | Yes | Yes | Yes | Yes | No | Yes | Yes | Yes | Yes |
| Teller/2023 | Yes | Yes | Yes | Yes | No | Yes | Yes | Yes | Yes |
| Balsak/2023 | Yes | Yes | Yes | No | No | Yes | Yes | Yes | Yes |
| Scardua‐Silva/2024 | Yes | Yes | Yes | Yes | No | Yes | Yes | Yes | Yes |
| Boito/2023 | Yes | Yes | Yes | Yes | No | Yes | Yes | Yes | Yes |
| Kausel/2024 | Yes | Yes | Yes | Yes | No | Yes | Yes | Yes | Yes |
| Deuter/2024 | Yes | Yes | Yes | Yes | No | Yes | Yes | Yes | Yes |
| Trufanov et al/2024 | Yes | Yes | Yes | Yes | No | Yes | Yes | Yes | Yes |
| Planchuelo-Gomez/2023 | Yes | Yes | Yes | Yes | No | Yes | Yes | Yes | Yes |
| Mishra 2024 | Yes | Yes | Yes | Yes | No | Yes | Yes | Yes | Yes |
| Lith 2024 | Yes | Yes | Yes | Yes | No | Yes | Yes | Yes | Yes |
